# Muscle Organoids Reveal Exercise‐Like Contractions Rapidly Promote Muscle Health Via Lamtor1's Signaling to Both AMPK and mTOR

**DOI:** 10.1002/advs.202505989

**Published:** 2025-10-07

**Authors:** Ziyue Yao, Zongmin Jiang, Xupeng Liu, Liping Zhang, Siyu Guo, Yu Chen, Lanfang Luo, Shilin Ma, Peng Wang, Ng Shyh‐Chang

**Affiliations:** ^1^ State Key Laboratory of Organ Regeneration and Reconstruction Institute of Zoology Chinese Academy of Sciences University of Chinese Academy of Sciences Beijing Institute for Stem Cell and Regenerative Medicine Beijing 100101 P. R. China; ^2^ School of Biological Engineering Zhuhai Campus of Zunyi Medical University Guangdong 519000 P. R. China

**Keywords:** 3D organoid, AMPK, exercise mimicry, Lamtor1, mouse models, mTOR, skeletal muscle

## Abstract

Exercise triggers molecular changes in skeletal muscles, but distinguishing immediate responses from secondary inter‐organ interactions in muscle biopsies remains challenging. Here, this study differentiates human embryonic stem cells (hESCs) into induced skeletal muscle (iMusc) cells to identify hypertrophic factors and generates a novel 3D human iMusc organoid model for studying the direct effects of exercise‐like contractions. Transcriptomics profiling reveals iMusc organoids rapidly induced genes associated with calcium signaling, p38/MAPK, EGF/ErbB, and NGF pathways within 1 h, mimicking exercise responses in vivo. Proteomics profiling and in vivo validation reveal rapid activation of both AMPK and mTORC1 signaling, partly through increased Lamtor1 levels, resolving a paradox in exercise biology. Human muscle biopsy analyses reveal Lamtor1 decreases with aging, and increases with exercise. In vivo and organoid experiments both confirm Lamtor1's role in mTORC1‐induced strength and AMPK‐induced lipid metabolism. Overall, this 3D iMusc organoid model provides insights into primary contraction‐induced changes and identifies Lamtor1 as a novel therapeutic target for exercise mimicry.

## Graphical abstract

This study develops a novel 3D human skeletal muscle organoid platform to study the immediate molecular effects of exercise‐like contractions. The model uncovers rapid proteomic and transcriptomic responses, resolving a fundamental paradox by demonstrating how exercise‐induced Lamtor1 coordinately activates both AMPK and mTORC1. Lamtor1 is a compelling target for therapeutic exercise mimicry.

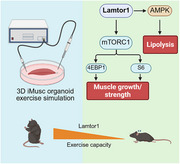

## 1 Introduction

Exercise involves skeletal muscle contractions that induce a variety of adaptive changes that, when repeated over time (*i*.*e*., physical training), result in molecular, structural, and functional remodeling of skeletal muscles.^[^ ^1^, ^2^, ^3^ ^]^ Changes in muscle hypertrophy and contraction not only affect mobility and respiration, but also general health and quality of life, as reflected in the prognosis for many chronic diseases of aging, such as sarcopenia, obesity, and type 2 diabetes.^[^ ^2^, ^4^ ^]^ While the molecular effects after exercise are well‐known, the immediate effects of skeletal muscle contraction have remained unclear.^[^ ^3^, ^5^ ^]^This has impeded our understanding of how best to phenocopy the full spectrum of benefits of muscle exercise.^[^ ^6^, ^7^ ^]^ Because of the inherent biomechanical and molecular differences between rodent and human muscles,^[^ ^8^, ^9^, ^10^, ^11^ ^]^ and the difficulties in obtaining biopsies of complete human muscle fibers that can contract long‐term in vitro,^[^ ^12^, ^13^, ^14^ ^]^ it has been challenging to study the early effects of muscle contractions.^[^ ^15^ ^]^ An alternative strategy is to leverage 3D organoid technology to build complete muscle organoids in vitro, and perform omics analyses soon after contractions.

Human muscle organoid construction, however, has not progressed beyond primary myoblasts‐ and fibrin‐based muscle organoids due to a variety of factors, including insufficient human muscle progenitors, immaturity in the differentiated myotubes and myofibers, inappropriate extracellular matrix (ECM) proteins to prevent delamination, and an inconsistent response after many cycles of electrical depolarization/stimulation.^[^ ^16^, ^17^, ^18^ ^]^ A good human muscle organoid should possess well‐aligned, hypertrophic and mature myofibers that are interspersed with muscle progenitors, fibroblasts, vascular cells, and collagen ECM, with long‐term contractility and mimicry of post‐exercise omics signatures in vivo. Such a human skeletal muscle organoid^[^ ^19^ ^]^ would allow us to simulate, measure, and perturb human muscle exercise in a controllable manner ex vivo.

By simulating neuronal action potentials with electrical pulse stimulation (EPS), such 3D organoid systems will permit a systematic dissection of exercise contraction‐induced mechanisms.^[^ ^20^ ^]^ Therefore, a mature 3D muscle organoid coupled with EPS may offer an electrophysiological platform that acts as an in vitro human muscle organoid model of exercise. While the vast majority of EPS studies have used long‐term (e.g., weeks‐long) regimens,^[^ ^18^ ^]^ short‐term stimulations (e.g., 1 h) could actually help to determine the changes in gene expression profiles that are directly and immediately activated by skeletal muscle contractions.^[^ ^21^ ^]^ Such data have been difficult to obtain via biopsies from muscle fibers in vivo.

At present, the hypothesized mechanisms of exercise‐induced muscle hypertrophy include the regulation of mTOR to promote muscle protein synthesis.^[^ ^22^, ^23^, ^24^, ^25^, ^26^, ^27^ ^]^ Unlike exercise‐induced AMPK signaling, which promotes catabolism, mTORC1 is a pro‐anabolic signaling complex that promotes cell growth by responding to growth factors (e.g., insulin, IGFs) and amino acids. The Lamtor/Ragulator (activator of mTORC1) regulatory complex participates in the sensing of amino acids and the activation of mTORC1.^[^ ^28^, ^29^ ^]^ Lamtor1 is directly responsible for anchoring the Ragulator complex to the membrane, where it mediates amino acid‐sensitive mTORC1 signaling. However, its role in mediating exercise‐induced muscle growth is not clear. Moreover, a long‐standing mystery in exercise biology is exactly how muscle contractions can both activate mTORC1 and AMPK,^[^ ^3^ ^]^ which is well‐known to inhibit growth factor‐dependent mTORC1‐S6K‐S6 signaling.^[^ ^30^ ^]^ While a study suggests contraction‐induced mTOR‐S6K‐S6 signaling is independent of Raptor‐S6K(T389) phosphorylation, other studies indicate it is sensitive to rapamycin and S6K(T421/S424) phosphorylation.^[^ ^31^, ^32^ ^]^ Thus, there must exist an alternative mechanism whereby exercise activates both mTORC1 and AMPK signaling, but this long‐sought mechanism has remained elusive.

Here, we used differentiation of human embryonic stem cells (hESCs) and high‐throughput drug screens to generate a hypertrophic 3D human muscle organoid system to study these questions. By combining short‐term EPS with transcriptomic and proteomic profiling, we were surprised to discover that a subset of classic gene/protein signatures associated with long‐term exercise were so rapidly induced in 3D human muscle organoids after stimulated contraction. Moreover, we uncovered how rapid induction of Lamtor1 protein shortly after muscle exercise can explain the paradoxical activation of both mTORC1 and AMPK, and we performed genetic perturbation experiments to confirm Lamtor1's importance for both muscle growth and lipid catabolism in vivo.

## 2 Results

### 2.1 Screening of Induced Human Skeletal Muscle Myotubes Reveals Hypertrophic Factors

**Figure 1:**
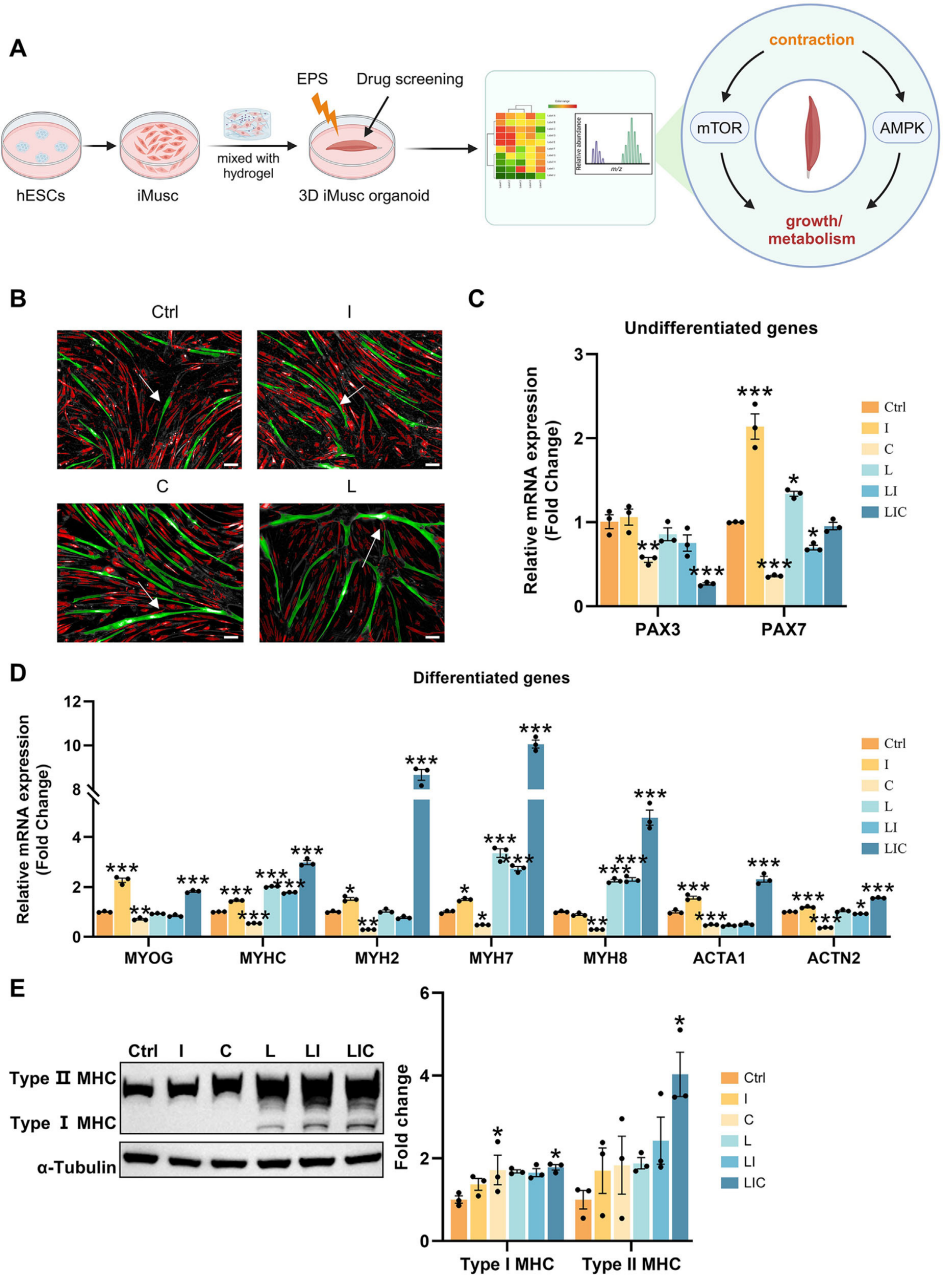
Induced skeletal muscle (iMusc) myoblasts and myotubes reveal the LIC drugs promote hypertrophy. A) A schematic diagram illustrates the experimental overview. 3D organoids were used in vitro to screen hypertrophic drugs. The organoids were subjected to electrical pulse stimulation (EPS) to elicit contractions mimicking physical exercise. B) Representative digital phase contrast (DPC) images show the estimated 3D volumes of human myotubes treated with different drugs. Ctrl (control), I (IGF‐1), C (calcitriol) and L (LY2157299). Myotubes are indicated in green, with white arrows pointing to representative examples; Scale bars, 100 µm. C, D) Quantitative RT‐PCR was performed to assess the gene expression of myogenic markers in iMusc myotubes treated with different drugs. Ctrl (control), C (calcitriol), I (IGF‐1), L (LY2157299), LI (LY2157299 + IGF‐1), LIC (LY2157299 + IGF‐1 + calcitriol). Genes analyzed: Paired box 3 (*PAX3*), Paired box 7 (*PAX7*), Myogenin (*MYOG*), Myosin heavy chain (*MYHC*), Myosin heavy chain 2 (*MYH2*), Myosin heavy chain 7 (*MYH7*), Myosin heavy chain 8 (*MYH8*), Skeletal muscle actin alpha 1 (*ACTA1*), actinin alpha 2 (*ACTN2*). Data are shown as means ± SEM from three independent experiments (*n* = 3). Statistical significance was determined by one‐way ANOVA followed by Tukey's post hoc test for multiple comparisons. *p <0.05, **p <0.01, ***p <0.001. E) Representative western blots of iMusc myotubes showing the expression of MHC (myosin heavy chain, MF20), a myogenic marker. α‐Tubulin served as the loading control (left). Quantification of MHC protein abundance normalized to α‐Tubulin, and fold changes relative to Ctrl are shown (right). Data are shown as means ± SEM from three independent experiments (*n* = 3). Statistical significance was determined by one‐way ANOVA followed by Tukey's post hoc test for multiple comparisons. *p <0.05, **p <0.01, ***p <0.001.

To obtain the large numbers of human skeletal muscle progenitors needed to culture 3D human myofibers with contractile function (**Figure** **1**A), we chose a myogenic directed differentiation strategy for hESCs with indefinite self‐renewal potential (Figure S1A, Supporting Information). Using a transgene‐free, chemically‐defined, step‐wise monolayer culture method, we progressively directed hESCs to undergo differentiation into induced skeletal muscle (iMusc) myoblasts and myotubes. First, after hESCs were withdrawn from mTESR1 media and triturated into small adherent clusters (Figure S1B, Supporting Information), a mixture of GSK3b, BMP, and TGFβR inhibitors was used to activate canonical Wnt signaling, suppress BMP signaling, and TGFβ signaling to induce somitic mesoderm (SM) formation over 4 days (Figure S1C, Supporting Information). Second, a mixture of FGF2 and GSK3b inhibitors was used to induce the specification of dermamyotome (DM) progenitors over 2 days (Figure S1D, Supporting Information). Third, HGF and IGF‐1 were utilized to induce DM progenitors to differentiate into skeletal muscle progenitors over 7–27 days (Figure S1E, Supporting Information), whereupon the PAX7+ muscle progenitors were passaged into a lower density monolayer of iMusc cells (Figure S1H, Supporting Information). Addition of serum and FGF2 induced the PAX7+ iMusc progenitors to commit into MyoD+ iMusc myoblasts over 27–32 days (Figure S1F,I, Supporting Information). Approximately 95% of cells were detected to express the CD56+/CD82+/CD201‐ phenotype in flow cytometry analysis at this time point (Figure S1J, Supporting Information). Finally, serum withdrawal and replacement with N2 media induced the MyoD+ iMusc myoblasts to undergo fusion, elongation, and differentiation into multinucleated myotubes (Figure S1G, Supporting Information) that were MYOG+ (Figure S1K, Supporting Information) and MHC+ (Figure S1L, Supporting Information). These well‐aligned mature myotubes are interspersed with quiescent PAX7+ progenitors, α‐SMA+ pericytes/stromal cells, and SOX9+ mesenchymal cells, all of which are Ki67‐negative (Figure S1M,N, Supporting Information). Collectively, the presence and spatial organization of these key cell types within the 3D organoids, including mature contractile myotubes (MHC+), quiescent muscle stem/progenitor cells (PAX7+), and supportive stromal cells (α‐SMA+, SOX9+), demonstrate the recapitulation of several hallmarks of human skeletal muscle tissue.

Using this large number of human iMusc myotubes, we performed a high‐throughput screen for drugs and proteins that induced post‐mitotic growth and maturation into hypertrophic myotubes.^[^ ^33^ ^]^ iMusc myoblasts at ≈90% confluency in 384‐well plates were differentiated and treated with ≈10 000 distinct drugs and proteins for 7–9 days. High‐content digital phase contrast (DPC) imaging combined with machine learning algorithms automatically quantified myotube hypertrophy parameters (3D volume, length, width, fusion index). Primary hits were defined as compounds inducing myotube volume >2 standard deviations above controls. From ≈10000 drugs and proteins, we found that the TGFβ inhibitor LY2157299 (L), IGF‐1 (I), and Calcitriol (C) had the strongest effect on myotubes’ hypertrophic maturation (Figure 1B). To obtain the maximum level of maturation, we proceeded to verify the effects of each molecule, individually and in combination, on myotube maturation at the mRNA and protein level. We found that C alone had the strongest effect in reducing gene expression of the myoblast transcription factors *PAX7* (Figure 1C), while LIC had the strongest effects in reducing the muscle progenitor marker *PAX3* (Figure 1C). For induction of the hypertrophy markers, LIC had the strongest effects on the myosin heavy chains (*MYHC, MYH2, MYH3, MYH7*, and *MYH8*) and skeletal muscle‐specific actin (*ACTA1* and *ACTN2*) expression (Figure 1D). IGF‐1 had the strongest effect on myogenic transcription factor *MYOG* (Figure 1D). Given that our earlier findings showed that C had a stronger effect on ribosomal translation than gene transcription,^[^ ^33^ ^]^ we proceeded to check for protein expression. Indeed, our results showed that C increased protein expression of the hypertrophy marker MHC, and the LIC combination had the strongest effects on MHC protein, especially the slow‐twitch MHC type I isoform (Figure 1E), which is usually only upregulated by exercise.^[^ ^34^, ^35^ ^]^

### 2.2 3D iMusc Organoids Show Enhanced Myogenesis Upon Exposure to the LIC Combination

**Figure 2:**
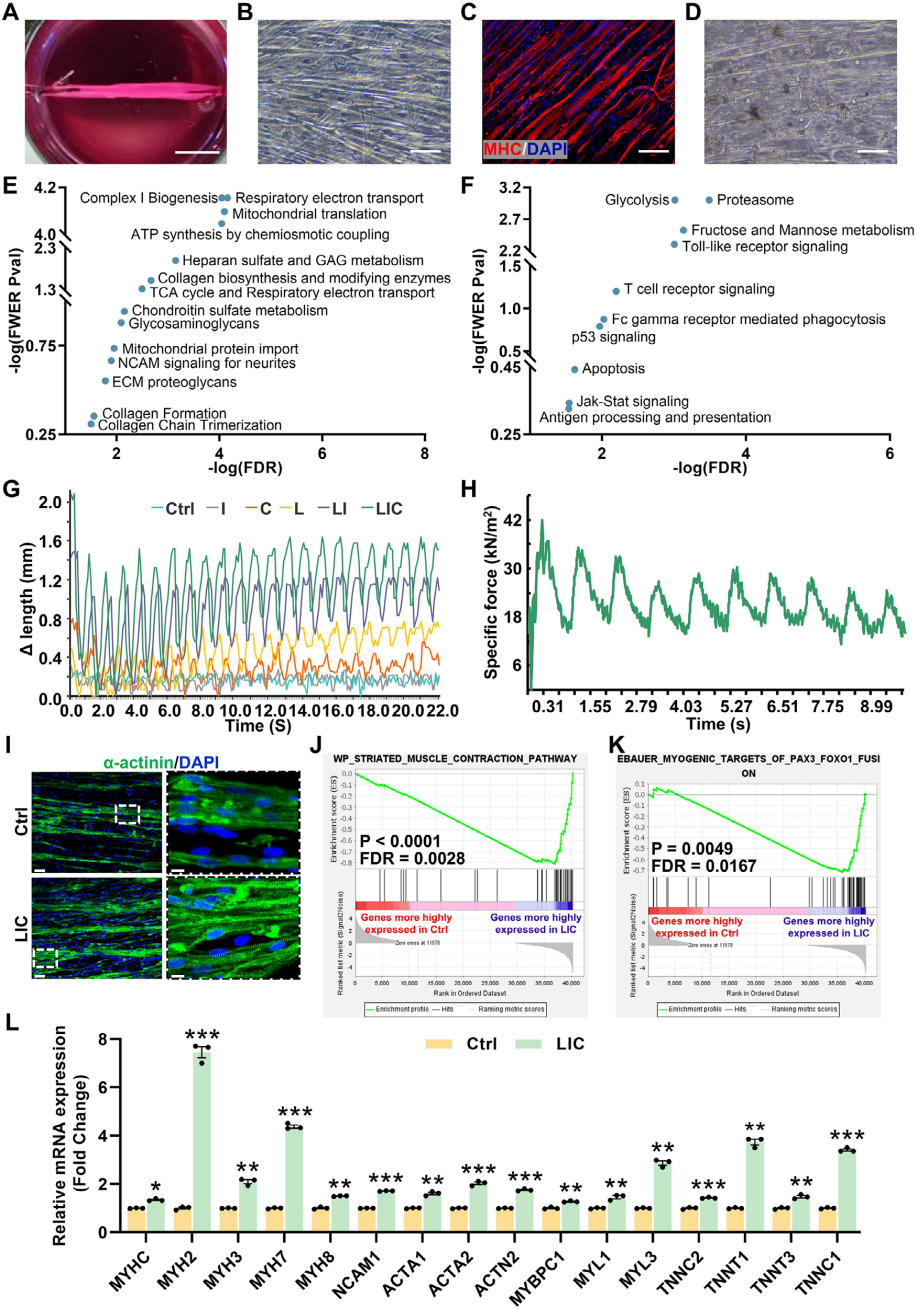
3D induced skeletal muscle (iMusc) organoids exhibit enhanced myogenesis upon exposure to the LIC drug combination. A) 2D cultured myoblasts were mixed with hydrogel to form 3D organoids anchored to a cured polydimethylsiloxane (PDMS). Scale bars, 1 cm. B) Self‐assembly and alignment of myotubes in a 3D iMusc organoid. Scale bars, 100 µm. C) Representative 3D reconstruction of longitudinal iMusc organoids immunostained for MHC (red) from three independent experiments. scale bars, 50 µm. D) Representative image of poorly aligned myotubes in 3D organoids made using human adult primary skeletal myoblasts (HSKM) from three independent experiments. Scale bars, 100 µm. E) PSEA using MSigDB: family‐wise error rate (FWER) p‐value versus false discovery rate (FDR) plot for signatures upregulated in 3D iMusc versus HSKM organoids. F) PSEA using MSigDB: FWER p‐value versus FDR plot for signatures downregulated in 3D iMusc versus HSKM organoids. G) Representative absolute length change (ΔLength) of 3D iMusc organoids under continuous EPS (1 Hz, 30 V, 10 ms pulse width, 1000 ms inter‐pulse intervals) with different drug treatments from three independent experiments. Ctrl (control), C (calcitriol), I (IGF‐1), L (LY2157299), LI (LY2157299 + IGF‐1), LIC (LY2157299 + IGF‐1 + calcitriol). H) Representative traces of specific twitch force (kN m^‐^^2^) from LIC‐treated 3D iMusc organoids under EPS (1 Hz, 30 V, 10 ms pulse width, 1000 ms inter‐pulse intervals, 20s duration) from three independent experiments. I) Representative longitudinal sections of Ctrl (top) and LIC‐treated (bottom) 3D iMusc organoids from three independent experiments, immunostained for α‐actinin (green) to visualize sarcomeres. Scale bars, 50 µm; enlarged view scale bars, 10 µm. J, K) Gene Set Enrichment Analysis (GSEA) plot for myogenesis gene sets upregulated in LIC‐treated versus Ctrl 3D iMusc organoids. L) Upregulation of myogenic markers and muscle contraction genes in LIC‐treated versus Ctrl 3D iMusc organoids validated by quantitative RT‐PCR. Genes analyzed: Myosin heavy chain 3 (*MYH3*), Neural cell adhesion molecule 1 (*NCAM1*), Smooth muscle actin alpha 2 (*ACTA2*), Myosin binding protein C1 (*MYBPC1*), Myosin light chain 1 (*MYL1*), Myosin light chain 3 (*MYL3*), Fast skeletal type troponin C2 (*TNNC2*), Slow skeletal type troponin T1 (*TNNT1*), Fast skeletal type troponin T3 (*TNNT3*), Slow skeletal and cardiac type troponin C1 (*TNNC1*). Data are shown as means ± SEM from three independent experiments (*n* = 3). Statistical significance was determined by two‐tailed Welch's *t*‐test for *MYH3*, *MYL3*, *TNNT1* and two‐tailed unpaired Student's *t*‐test for other genes. *p <0.05, **p <0.01, ***p <0.001.

To definitively test for myotube maturation into hypertrophic myofibers, we utilized 3D cultures of iMusc organoids. Dissociation of millions of the above‐generated human iMusc myoblasts and myotubes, followed by seeding onto a fibrin‐Matrigel hybrid scaffold, permitted a self‐organization of the cells into a 3D iMusc organoid that was ≈3 cm long (**Figure** **2**A). We used light microscopy to examine the construct and found that the 3D iMusc organoid contained well‐aligned human myotubes (Figure 2B), whereas light sheet fluorescence microscopy revealed a dense 3D array of well‐aligned MHC+ multinucleated myotubes within the iMusc organoid (Figure 2C). Immunofluorescence staining of Dystrophin was performed to evaluate the cross‐sectional area (CSA) of the iMusc organoids (Figure S2A, Supporting Information). In comparison, when we used human adult primary skeletal myoblasts (HSKM) or rejuvenated LTS myoblasts,^[^ ^36^ ^]^ the formation efficiency of self‐aligned 3D organoids was poor (Figure 2D). These results suggest that iMusc progenitors can more effectively mimic early embryonic development in laying down and sculpting the ECM microenvironment needed to support the self‐organization of entire 3D myofibers, compared to HSKM, which primarily mediate localized muscle regeneration upon small injuries. Indeed, TMT‐based quantitative proteomics and MSigDB‐based protein set enrichment analysis (PSEA)^[^ ^37^, ^38^, ^39^, ^40^ ^]^ showed that iMusc organoids were enriched with higher levels of ECM proteins associated with collagen biosynthesis and glycosaminoglycans (GAGs), such as heparan sulfate and chondroitin, as well as mitochondrial proteins associated with the respiratory electron transport chain and the TCA cycle, compared to HSKM(Figure 2E). In contrast, HSKM‐derived 3D organoids were enriched with higher levels of glycolysis enzymes, proteasome subunits, inflammatory‐immune signaling proteins, and p53‐apoptosis signaling proteins (Figure 2F). Direct comparison further revealed that iMusc progenitors exhibited similar fusion indices but >1.5‐fold faster proliferation than HSKM in vitro (Figure S1O,P, Supporting Information). Critically, iMusc‐derived myotubes maintained structural integrity for >1‐week post‐differentiation, while HSKM‐derived myotubes underwent complete detachment within 96 h. These differences mechanistically explain the superior contractility and stability of iMusc organoids. Collectively, iMusc organoids demonstrate greater maturity, regarding their ECM, mitochondria, and resistance to inflammation, atrophy, and apoptosis.

To test the level of maturation induced by the LIC drugs, we functionally examined the contractility of the 3D iMusc organoids upon electrical stimulation and depolarization to induce muscle contractions every second (1 Hz). We found that the LIC combination, as compared to monotreatments or dual L+I treatment, induced the highest level of endurance, with similarly large amplitudes even after 20 contraction cycles (Figure 2G). In contrast, the single or dual L+I treatments either showed gradual degradation in amplitude or, like the vehicle control, could only maintain very small amplitudes in length contractions (Figure 2G). We also measured the specific twitch force generated during these contractions, and found that the LIC combination induced the largest twitch force (≈41 mN mm^−2^ or kN m^−2^) at the first contraction, and it maintained ≈23 mN mm^−2^ or kN m^−2^ at the 9th contraction (Figure 2H; Figure S2B, Supporting Information). In contrast, the other treatments could only produce smaller specific twitch forces (Figure S2C, Supporting Information).

To examine the origins of the higher specific twitch force induced by LIC, we performed high‐resolution immunofluorescence confocal microscopy and transmission electron microscopy imaging of the 3D iMusc organoids. By α‐actinin immunostaining and transmission electron microscopy imaging, we found that LIC‐treated iMusc organoids had the most abundant and well‐developed α‐actinin+ striations, Z‐discs, and mitochondria (Figure 2I; Figure S3, Supporting Information), whereas the other treatments led to fewer mitochondria and a more diffuse distribution of α‐actinin (Figure S3, Supporting Information), indicating that LIC‐treated iMusc organoids did develop higher levels of maturation in their myofibrillar and organellar apparatus.

To confirm that LIC treatment only enhanced the skeletal myogenesis program and not other developmental programs, we performed RNA‐sequencing on the LIC‐treated iMusc organoids. By gene set enrichment analysis (GSEA) we found that the LIC combination significantly enhanced several transcriptional signatures of myogenesis, including signatures for Striated Muscle Contraction (Figure 2J) and PAX3 gene targets in myogenesis (Figure 2K). Many of these gene targets were validated by quantitative RT‐PCR, including *MYHC*, *MYH2*, *MYH3*, *MYH7*, *MYH8*, *NCAM1*, *ACTA1*, *ACTA2*, *ACTN2*, *MYBPC1*, *MYL1, MYL3*, *TNNC2*, *TNNT1*, *TNNT3*, and *TNNC1* (Figure 2L). In addition, GSEA also revealed that the LIC combination induced IGF signaling and suppressed TGFβ signaling, as expected of the I and L drugs, respectively (Figure S4, Supporting Information). Moreover, the LIC combination also synergistically upregulated transcriptional signatures for Ribosomes, Mitochondrial oxidative phosphorylation, and Robo‐Slit signaling (Figure S4, Supporting Information), and downregulated transcriptional signatures for Hypoxia and Inflammation (Figure S5, Supporting Information), as expected of hypertrophic and metabolically efficient myofibers that are ready to attract innervation, and no longer subject to the injury‐responsive hypoxia and inflammation responses that are characteristic of muscle progenitors.^[^ ^36^ ^]^

### 2.3 3D iMusc Organoids Rapidly Induce Exercise‐Associated mRNA Signatures After Depolarization

**Figure 3:**
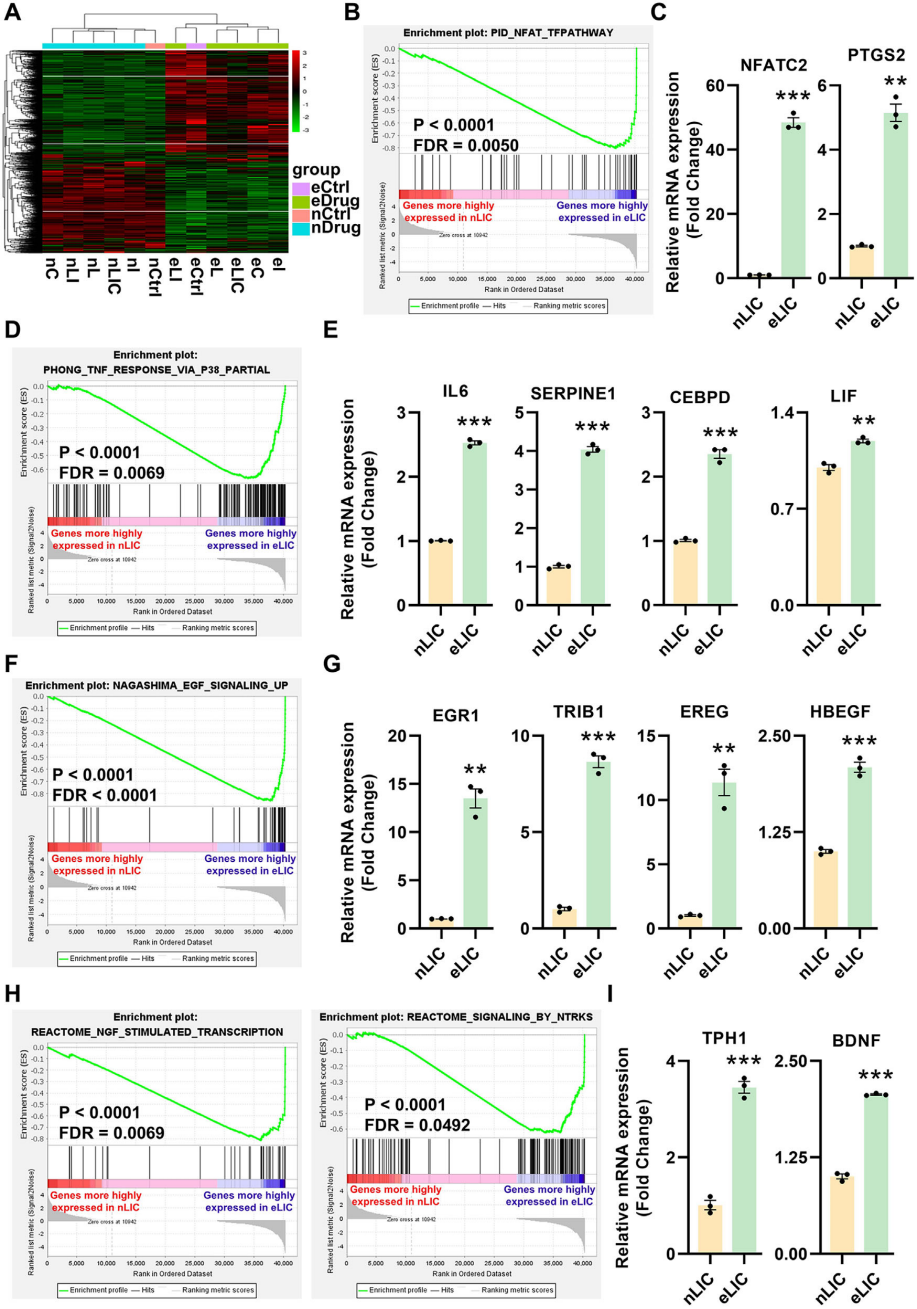
Electrical depolarization‐induced contractions rapidly activate exercise‐associated transcriptomic signatures in 3D induced skeletal muscle (iMusc) organoids. A) Hierarchical clustering of differentially expressed genes between electrical stimulated (e) and non‐stimulated (n) groups. Contractions in e‐group iMusc organoids were induced by 5‐min continuous EPS (1 Hz, 30 V, 10 ms pulse width, 1000 ms inter‐pulse intervals). Groups: Ctrl and drug treatments (C/I/L/LI/LIC). B) GSEA plot showing upregulated NFAT signaling pathway signatures in eLIC versus nLIC organoids. C) Quantitative RT‐PCR validation of upregulated Ca^2+^ and NFAT signaling pathway genes in eLIC versus nLIC organoids. Genes: Nuclear factor of activated T cell 2 (*NFATC2*), Prostaglandin‐endoperoxide synthase 2 (*PTGS2*). Data are shown as means ± SEM from three independent experiments (*n* = 3). Statistical significance was determined by two‐tailed Welch's *t*‐test. *p <0.05, **p <0.01, ***p <0.001. D, E) GSEA plot and quantitative RT‐PCR validation of upregulated p38 MAPK signaling pathway signatures in eLIC versus nLIC organoids. Genes: Interleukin 6 (*IL‐6*), Serpin Family E Member 1 (*SERPINE1*), CCAAT Enhancer Binding Protein Delta (*CEBPD*), Leukemia Inhibitory Factor (*LIF*). Data are shown as means ± SEM from three independent experiments (*n* = 3). Statistical significance was determined by two‐tailed Welch's *t*‐test for *IL‐6* and two‐tailed unpaired Student's *t*‐test for other genes. *p <0.05, **p <0.01, ***p <0.001. F, G) GSEA plot and quantitative RT‐PCR validation of upregulated EGFR signaling pathway signatures in eLIC versus nLIC organoids. Genes: Early growth response 1 (*EGR1*), Tribbles pseudokinase 1 (*TRIB1*), Epiregulin (*EREG*), Heparin binding EGF like growth factor (*HBEGF*). Data are shown as means ± SEM from three independent experiments (*n* = 3). Statistical significance was determined by two‐tailed Welch's *t*‐test for *EREG*, *EGR1* and two‐tailed unpaired Student's *t*‐test for other genes. *p <0.05, **p <0.01, ***p <0.001. H, I) GSEA plots and quantitative RT‐PCR validation of upregulated NGF and NTR signaling pathway signatures in eLIC versus nLIC organoids. Genes: Tryptophan hydroxylase 1(*TPH1*), Brain derived neurotrophic factor (*BDNF*). Data are shown as means ± SEM from three independent experiments (*n* = 3). Statistical significance was determined by two‐tailed unpaired Student's *t*‐test. *p <0.05, **p <0.01, ***p <0.001.

Having demonstrated that our LIC‐optimized 3D iMusc organoids represent hypertrophic, mature and contractile myofibers, we then used this model system to answer questions regarding human exercise that previously could not be easily tackled by skeletal muscle samples in vivo or ex vivo. Notably, we examined the primary transcriptomic changes in human skeletal muscles that are rapidly and directly induced by muscle depolarization and exercise contractions, before secondary changes are induced in other non‐muscle cells and tissues. We performed RNA‐sequencing on all treated 3D iMusc organoids, with and without electrical stimulation/depolarization‐induced contractions. Extant literature suggest that signals like doxycycline take at least 4 h to significantly induce gene transcription.^[^ ^41^, ^42^, ^43^ ^]^ Unexpectedly, our results showed that electrical stimulation/depolarization‐induced contractions rapidly induced major changes in the human muscle transcriptome within 1 h, major changes that even exceed the differences due to hypertrophic maturation (**Figure** **3**A). Changes induced by electrical stimulation/depolarization‐induced contractions overlap significantly across iMusc organoid samples (Figure S6, Supporting Information), so we focused on LIC‐treated organoids to understand the early, primary transcriptional changes in our most mature and hypertrophic organoid model.

By GSEA we found that electrical stimulation/depolarization rapidly upregulated the calcium‐calcineurin‐NFAT signaling pathway in iMusc organoids (Figure 3B), as expected of depolarized myofibers.^[^ ^44^, ^45^, ^46^ ^]^ Many of the gene targets were validated by quantitative RT‐PCR, including *NFATC2* and *PTGS2* (Figure 3C). In addition, by GSEA we found that electrical stimulation directly upregulated the p38 MAPK signaling pathway in iMusc organoids (Figure 3D). Previous studies have also shown that intense exercise can rapidly induce p38 MAPK signaling in the skeletal muscles,^[^ ^47^, ^48^, ^49^ ^]^ but it was unclear if it was due to a growth factor signal acting in a paracrine/endocrine manner. Validation of the p38 targets directly induced by electrical stimulation further revealed that they included the expression of the myokines *IL‐6* and *LIF*, a pro‐regenerative serpin E, as well as the transcription factor *CEBPD* (Figure 3E).

Furthermore, by GSEA we found that electrical stimulation directly upregulated the epidermal growth factor (EGF)‐ErbB signaling pathway in iMusc organoids (Figure 3F). Previous studies have also shown that intense exercise can induce ErbB signaling in the skeletal muscles,^[^ ^50^, ^51^ ^]^ but it was also unclear if it was an early primary or secondary response to exercise. Validation of the ErbB signaling markers further revealed that they included the EGF‐like factors and myokines *EREG* and *HBEGF*, the kinase *TRIB1*, as well as the transcription factor *EGR1* (Figure 3G). Finally, by GSEA we found that electrical stimulation directly upregulated the nerve growth factor (NGF) signaling pathway in iMusc organoids (Figure 3H). Previous studies have also shown that exercise can eventually induce NGF signaling in the skeletal muscles,^[^ ^52^, ^53^ ^]^ but it was assumed to be due to innervation and paracrine signaling among the neurons in muscle fibers, although the BioGPS and Gtex databases show that it can also be expressed in non‐neuronal cells. Validation of the NGF signaling markers in iMusc organoids further revealed that they included the myokine *BDNF* and the serotonin biosynthetic enzyme *TPH1* (Figure 3I).

Taken together, these transcriptome results demonstrate that our 3D iMusc organoids biomimic many molecular features of human muscles after exercise while clarifying that depolarization‐induced contractions can directly and rapidly induce calcium‐NFAT, p38 MAPK, EGF‐ErbB, and NGF signaling in human muscle cells, while in the process upregulating the expression of many well‐known myokines, such as *IL‐6*, *LIF*, *EREG*, *HBEGF*, and *BDNF*.

### 2.4 3D iMusc Organoid Contractions Rapidly Induce Exercise‐Like Protein Changes, Such as Lamtor1

**Figure 4:**
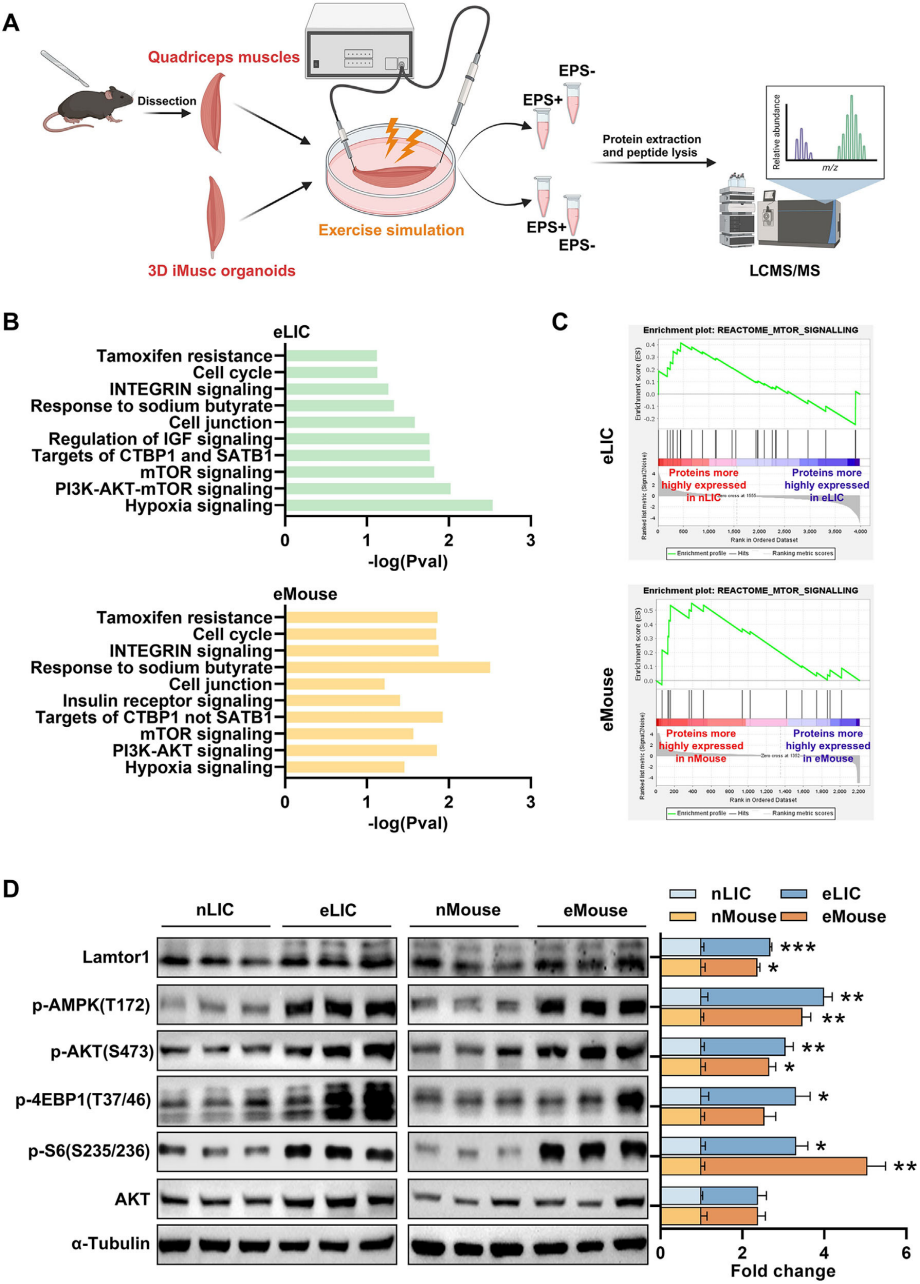
Depolarization‐induced contractions rapidly activate exercise‐associated proteomic changes in pure muscle cells, simultaneously engaging both AMPK and mTOR pathways. A) Schematic workflow for proteomic analysis of quadriceps muscle tissue isolated from C57BL/6 mice (*n* = 4) and 3D eLIC‐treated induced skeletal muscle (iMusc) organoids (*n* = 4) subjected to 1 h of continuous electrical stimulation (EPS+) or non‐stimulated controls (EPS‐). Protein extraction and tryptic digestion were followed by LC‐MS/MS and data analysis. B) Bar plots derived from quantitative LC‐MS/MS proteomics showing ‐log(P‐values) of the top overlapping PSEA signatures enriched in eLIC‐treated iMusc organoids (relative to nLIC controls, top) and eMouse quadriceps muscles (relative to nMouse controls, bottom). C) PSEA plots generated via LC‐MS/MS proteomics showing upregulation of mTOR signaling signatures in eLIC‐treated iMusc organoids (top) and eMouse quadriceps muscles (bottom). D) Representative western blots of mTOR and AMPK signaling proteins in LIC‐treated iMusc organoids (left) and mouse quadriceps muscles (right). α‐tubulin served as the loading control. Protein abundance was normalized to α‐tubulin, and fold changes are shown as means + SEM from three biological replicates (*n* = 3). Statistical significance determined by two‐tailed unpaired Student's *t*‐test for all proteins. *p <0.05, **p <0.01, ***p <0.001.

Given that transcriptional changes require changes in transcription factor expression or activity, it is likely that electrical stimulation first affects protein expression and phosphorylation, before regulating mRNA expression. To verify if our 3D iMusc organoids also biomimic the key primary protein changes seen in skeletal muscles after exercise in vivo, we compared its proteome to the proteome of mouse quadriceps muscles, both of which were stimulated to contract using electrical depolarization (**Figure** **4**A). By PSEA of the differentially abundant proteins observed after 1 h of electrically stimulated contractions we found that the top overlapping signatures included mTOR signaling and insulin‐PI3K(‐mTOR) signaling (Figure 4B,C), consistent with exercise‐induced insulin sensitivity, followed by signatures in gap junctions, integrins, cell cycle, and the downregulation of Hypoxia targets, CTBP1 targets, and Butyrate HDAC inhibitor targets (Figure 4B).

To verify the observation on mTOR signaling, we performed semi‐quantitative Western blot analysis of the iMusc organoids and ex vivo mouse muscles, with and without electrical stimulation. As expected, and as reported before,^[^ ^54^, ^55^ ^]^ both the iMusc organoids and ex vivo mouse myofibers showed increases in phospho‐AMPK after contractions (Figure 4D). When examined for mTOR signaling components, both iMusc organoids and ex vivo mouse myofibers showed higher phospho‐AKT, phospho‐4EBP, and phospho‐S6 levels, with a less marked increase in phospho‐4EBP (Figure 4D). Such results indicate that both AMPK and mTORC1 are activated in the muscle fibers simultaneously, which is a paradoxical signaling phenomenon unique to physical exercise, given the mutual inhibition^[^ ^56^, ^57^ ^]^ between pro‐catabolic AMPK signaling, which promotes mitochondrial fatty acid oxidation after physical activity,^[^ ^58^, ^59^, ^60^, ^61^ ^]^ and pro‐anabolic mTORC1 signaling, which is needed to promote muscle strength after physical activity.^[^ ^22^, ^56^, ^57^, ^62^ ^]^ Our Western blot results further illustrate how this might be possible, by showing an increase in the amino acid‐sensitive Lamtor/Ragulator (one of our top hits in proteomics profiling) that can activate either AMPK or mTORC1 signaling and thus phosphorylation of 4EBP and S6 (Figure 4D). Thus, characterization of 3D iMusc organoids shortly after exercise‐like contractions revealed that the rapid and paradoxical activation of both AMPK and mTOR signaling could be due to rapid Lamtor1 upregulation by exercise.

### 2.5 Rapid Induction of Muscle Lamtor1 is Needed for mTOR‐Induced Strength and AMPK‐Induced Lipid Metabolism After Exercise

**Figure 5:**
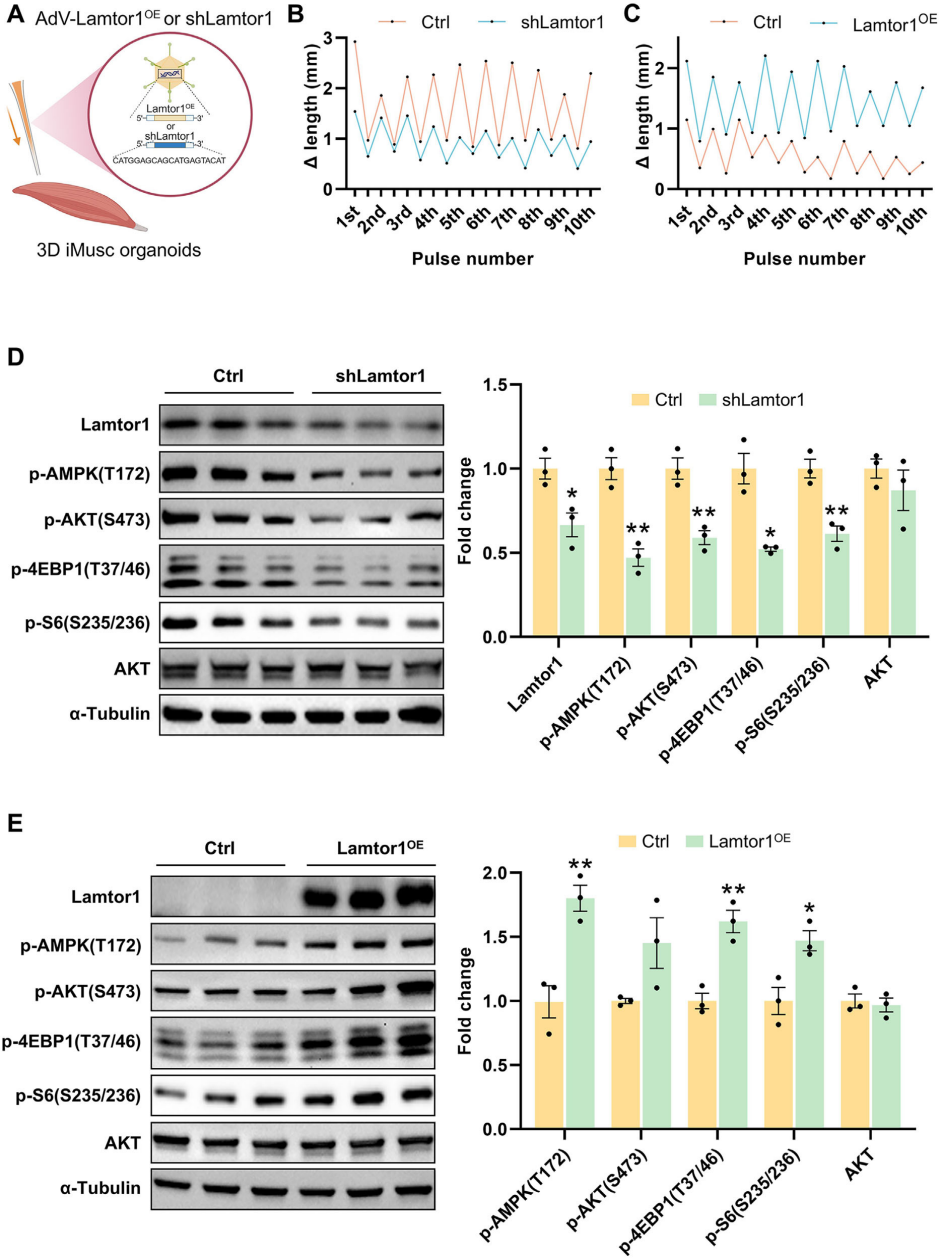
Gain‐ and loss‐of‐function of Lamtor1 modulate AMPK and mTORC1 signaling in 3D induced skeletal muscle (iMusc) organoids. A) Schematic illustration of experimental procedure for delivering AdV‐shLamtor1 or AdV‐Lamtor1^OE^into 3D iMusc organoids. B) Representative absolute length change (ΔLength) of AdV‐Ctrl and AdV‐shLamtor1 3D iMusc organoids under continuous EPS (1 Hz, 30 V, 10 ms pulse width, 1000 ms inter‐pulse interval) from three independent experiments with similar results. C) Representative absolute length change (ΔLength) of AdV‐Ctrl and AdV‐Lamtor1^OE^3D iMusc organoids under continuous EPS (1 Hz, 30 V, 10 ms pulse width, 1000 ms inter‐pulse interval) from three independent experiments with similar results. D) Representative western blots of mTOR and AMPK signaling proteins in AdV‐Ctrl (control; left) and AdV‐shLamtor1 (right) iMusc organoids. α‐tubulin served as the loading control. Protein abundance was normalized to α‐tubulin, and fold changes are shown as means ± SEM from three biological replicates (*n* = 3). Statistical significance determined by two‐tailed unpaired Student's *t*‐test for all proteins. *p <0.05, **p <0.01, ***p <0.001. E) Representative western blots of mTOR and AMPK signaling proteins in AdV‐Ctrl (control; left) and AdV‐Lamtor1^OE^(right) iMusc organoids. α‐tubulin served as the loading control. Protein abundance was normalized to α‐tubulin, and fold changes are shown as means ± SEM from three biological replicates (*n* = 3). Statistical significance determined by two‐tailed Welch's *t*‐test for p‐4EBP1 and two‐tailed unpaired Student's *t*‐test for all other proteins. *p <0.05, **p <0.01, ***p <0.001.

**Figure 6:**
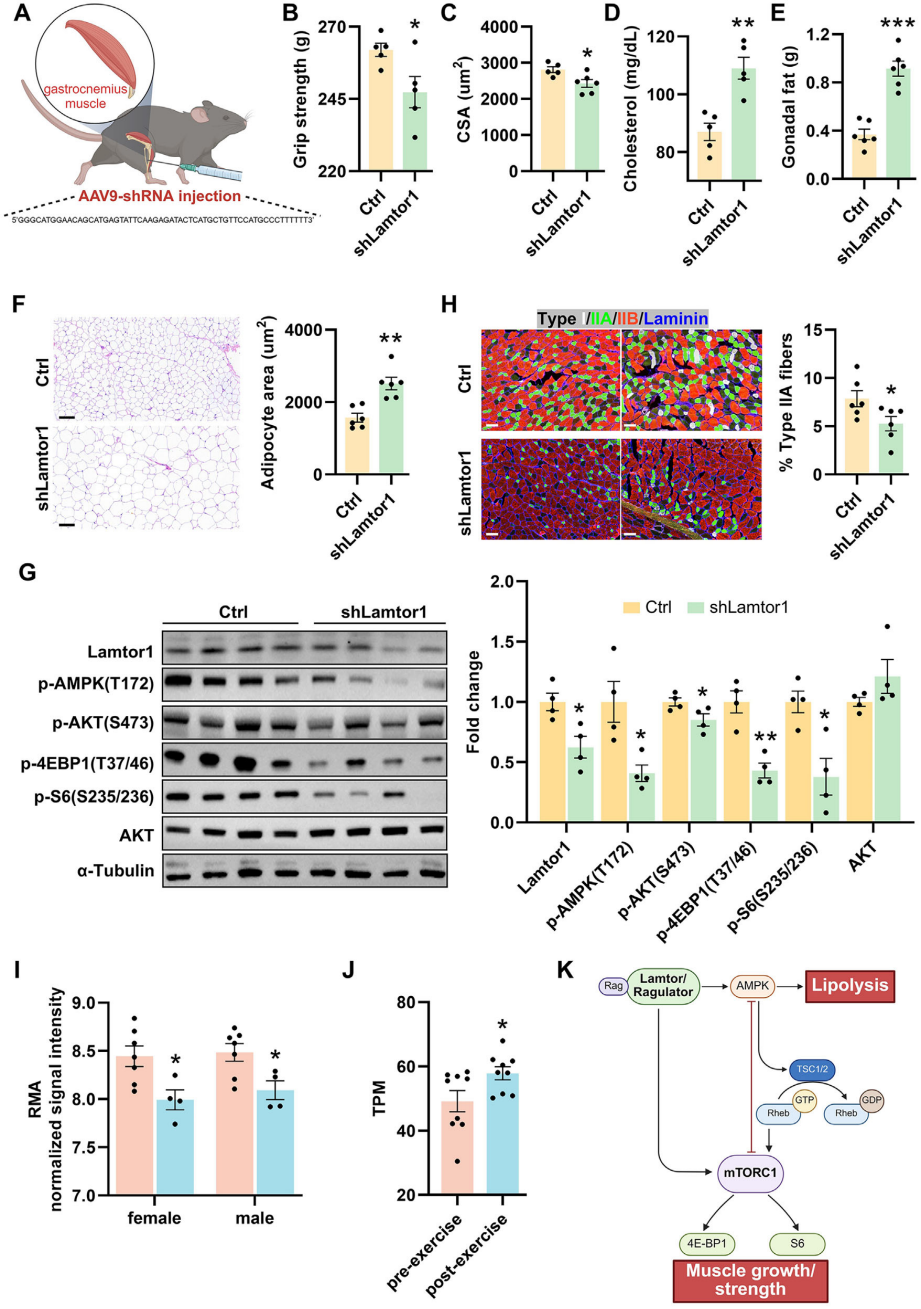
Lamtor1 knockdown mice exhibit reduced skeletal muscle strength and increased fat accumulation. A) Schematic illustration of experimental procedure for delivering AAV9‐shRNA into the gastrocnemius muscles of C57BL/6 mice. B) Grip strength measurements in Ctrl (control) and the shLamtor1 mice. Data are shown as means ± SEM (*n* = 5). Statistical significance was determined by two‐tailed unpaired Student's *t*‐test. *p <0.05, **p <0.01, ***p <0.001. C) Quantitative analysis of cross‐sectional area (CSA) in quadriceps muscle of Ctrl (*n* = 5) and the shLamtor1 (*n* = 6) mice. Data are shown as means ± SEM. Statistical significance was determined by two‐tailed unpaired Student's *t*‐test. *p <0.05, **p <0.01, ***p <0.001. D) Serum cholesterol levels in Ctrl and the shLamtor1 mice after fasting for 16 hs. Data are shown as means ± SEM (*n* = 5). Data are shown as means ± SEM. Statistical significance was determined by two‐tailed unpaired Student's *t*‐test. *p <0.05, **p <0.01, ***p <0.001. E) Gonadal fat mass in Ctrl and the shLamtor1 mice. Data are shown as means ± SEM (*n* = 6). Data are shown as means ± SEM. Statistical significance was determined by two‐tailed unpaired Student's *t*‐test. *p <0.05, **p <0.01, ***p <0.001. F) Representative images of gonadal fat and quantification of adipocyte area. Scale bars, 100 µm. Data are shown as means ± SEM (*n* = 6). Statistical significance was determined by two‐tailed unpaired Student's *t*‐test. *p <0.05, **p <0.01, ***p <0.001. G) Representative western blots of mTOR and AMPK signaling proteins in Ctrl (left) and the shLamtor1 (right) mice. α‐tubulin served as the loading control. Protein abundance was normalized to α‐tubulin, and fold changes are shown as means ± SEM (*n* = 4). Statistical significance was determined by two‐tailed unpaired Student's *t*‐test for all proteins. *p <0.05, **p <0.01, ***p <0.001. H) Immunostaining of gastrocnemius muscles obtained from Ctrl and the shLamtor1 mice for detection of type I (white), type IIA (green) and type IIB (red) myofibers (left). Scale bars, 100 µm. Quantification of the area percentage of type IIA myofibers (right). Data are shown as means ± SEM (n = 6). Statistical significance was determined by two‐tailed unpaired Student's *t*‐test. *p <0.05, **p <0.01, ***p <0.001. I) GDS4858: (young [19‐28 yr] versus old [65‐76 yr] muscle biopsies). This study included 42 individuals (both sexes; no resistance training ≥1 year prior to biceps brachii biopsy). Statistical significance determined by two‐tailed unpaired Student's *t*‐test. J) GSE164081 (post‐exercise versus pre‐exercise). Ten male amateur endurance‐trained athletes (median age 32 years [IQR 30–36]; weight 75 kg [IQR 71–78]) were included. Statistical significance determined by two‐tailed Mann‐Whitney test. K) Schematic representation of the signaling axis linking Lamtor, mTOR and AMPK.

To confirm that the rapid upregulation of Lamtor1 protein in both mouse and human skeletal muscles after exercise, has a functional role in mediating the physiological effects of mTOR signaling and AMPK signaling after exercise, we used shRNAs to knockdown Lamtor1 (**Figures** **5**A and **6**A). After optimizing the shRNA sequences with adenoviruses (Figure S7A,B, Supporting Information), several iMusc organoids were infected with AdV‐Ctrl and ‐shLamtor1, respectively. AdV‐shLamtor1‐treated organoids remained viable with no discernible structural damage, though amplitudes in length contractions were slightly reduced versus AdV‐Ctrl (Figure 5B, Video S1). A semi‐quantitative Western blot showed that AdV‐shLamtor1 iMusc organoids had lower levels of phospho‐AMPK shortly after contraction (Figure 5D). AdV‐shLamtor1 iMusc organoids also had lower levels of the mTORC1 targets phospho‐S6 and phospho‐4EBP1, shortly after contraction (Figure 5D).^[^ ^22^, ^62^ ^]^ To comprehensively assess the functional role of Lamtor1 upregulation and directly address its therapeutic potential, we next employed adenovirus‐mediated overexpression of Lamtor1 (AdV‐Lamtor1^OE^) in iMusc organoids (Figure 5A; Figure S7C,D, Supporting Information). AdV‐Lamtor1^OE^organoids exhibited a higher amplitude in length contractions versus AdV‐Ctrl organoids (Figure 5C), implying enhanced contractile capacity. A semi‐quantitative Western blot showed that AdV‐Lamtor1^OE^iMusc organoids had higher levels of phospho‐AMPK shortly after contraction (Figure 5E). AdV‐Lamtor1^OE^iMusc organoids also had higher levels of the mTORC1 targets phospho‐S6 and phospho‐4EBP1, shortly after contraction (Figure 5E). These data confirm that Lamtor1 upregulation amplifies AMPK‐driven contractile response signaling in human muscle models, while concurrently enhancing mTORC1 signaling targets (phospho‐S6 and phospho‐4EBP1). Together with the knockdown results, these findings support a role for Lamtor1 as a bifunctional signaling scaffold capable of bidirectionally regulating both AMPK and mTORC1 pathways. Based on the rapid short‐term effects of AdV‐shLamtor1 iMusc organoids and the complementary enhancing effects of AdV‐OE‐Lamtor1^OE^, we wondered whether knockdown of Lamtor1 would have the same effects in vivo. However, one reason we had to use 3D organoids to dissect and unveil this rapid, short‐term exercise mechanism is because it is extremely difficult to controllably induce mice to contract vigorously after an extended period of whole‐body immobility. Instead, we chose to test if we could achieve more stable ablation of exercise‐induced AMPK, mTORC1, and changes in physical indicators and exercise capacity, after long‐term knockdown of Lamtor1. After optimizing the muscle‐specific AAV9‐shRNA sequence and intramuscular dosage (Figure S7E–G, Supporting Information), 10 physically active mice per group were administered with intramuscular shLamtor1 or control AAV9‐shRNAs (Figure S8A, Supporting Information). Comprehensive toxicity assessment confirmed safety (S8B–I). Despite similar endurance in hanging and running times (Figure S8J,K, Supporting Information), the AAV9‐shLamtor1 mice displayed significantly lower limb muscle grip strength and myofiber CSA (Figure 6B,C), relative to the control mice. Moreover, the AAV9‐shLamtor1 mice exhibited significantly elevated fed blood glucose and total serum cholesterol (Figure 6D; Figure S8L, Supporting Information), gonadal fat mass and adipocyte area (Figure 6E,F), than the control mice. A semi‐quantitative Western blot showed that AAV9‐shLamtor1 mice’ muscles had the same trend of changes as AdV‐shLamtor1 iMusc organoids (Figure 6G), which had lower levels of phospho‐AMPK and the mTORC1 targets phospho‐S6 and phospho‐4EBP1 (Figure 6G). Lamtor1 knockdown significantly reduced type IIA fibers, with non‐significant decreases in type I fibers and increases in type IIB fibers (Figure 6H; Figure S8M,N, Supporting Information), indicating its selective regulation of oxidative phenotypes. Analyses of Lamtor1 expression levels in human biceps brachii muscles biopsies (42 individuals: 19–28y and 65–76y, both sexes, no resistance training ≥1 year; Figure 6I) showed that older subjects (65–76y) had significantly lower Lamtor1 than younger subjects (19–28y), while young male participants that underwent intermittent exercise training had significantly higher Lamtor1 RNA expression (*n* = 10, age 32y [interquartile range, 30–36y]; Figure 6J). Taken together, our bidirectional genetic manipulations in vitro (knockdown and overexpression) combined with mouse experimental data and human data analyses revealed that the Lamtor1 induced by skeletal muscle exercise is needed for mTOR‐induced anabolic growth and AMPK‐induced lipid catabolism in vivo, and is relevant to human muscle physiology.

## 3 Discussion

In this study, we obtained large numbers of hESC‐derived muscle progenitors to construct contractile 3D human iMusc organoids that resemble exercising muscles of in vivo origin. This model uniquely recapitulates skeletal muscle development via self‐organization, achieving unprecedented >3 cm length (Figure 2A), sarcomere assembly (Figure S3, Supporting Information), ≈41 kN m^−^^2^ twitch specific force (Figure 2H), and contains a small number of PAX7+ stem cells as well as SMA+/SOX9+ stroma (Figure S1M, Supporting Information), thereby forming an endogenous cell diversity that recapitulates developmental myogenesis.^[^ ^63^ ^]^ Our iMusc organoids could be used to screen for pro‐muscle growth drugs and combinations thereof, as shown in our discovery of the LIC combination of hypertrophic factors, as a proof‐of‐principle. Mechanistically, LIC synergistically enhances ribosomal activity (C), activates protein synthesis via PI3K/AKT/mTOR (I), and alleviates TGFβ/Smad‐mediated (L) transcriptional repression of key myogenic regulatory factors.^[^ ^64^ ^]^ Moreover, our iMusc organoids revealed both previously known and previously unknown signatures of transcriptomic and proteomic changes that emerge shortly after exercise ex vivo. Although complete and fully contractile human myofiber tissues (not just a muscle biopsy) are not easy to obtain from the clinic, we did use complete mouse quadriceps to confirm that our iMusc organoids phenocopy the proteomic changes in mTOR signaling, mitochondrial, and neuromuscular junction proteins^[^ ^65^, ^66^ ^]^ that are observed in contracting muscles after 1 h of exercise. Moreover, our findings resolved a long‐standing mystery^[^ ^3^, ^67^ ^]^ of how exercise‐induced mTOR signaling (anabolic) can paradoxically coexist with the exercise‐induced AMPK signaling (catabolic), despite their mutual inhibition,^[^ ^57^, ^68^, ^69^, ^70^, ^71^, ^72^ ^]^ by inducing Lamtor1/Ragulator, which participates in both signaling pathways (Figure S8O, Supporting Information).

Extant literature suggests that signals like doxycycline take at least 4 h to significantly induce gene transcription.^[^ ^41^, ^42^, ^43^ ^]^ Unexpectedly, our results showed that electrical stimulation/depolarization‐induced contractions rapidly induced major changes in the human muscle transcriptome within 1 h, major changes that even exceed the differences due to weeks of LIC treatment. Because the changes induced by electrical stimulation/depolarization‐induced contractions overlap significantly across organoid samples, we focused on LIC‐treated organoids to understand the primary transcriptional changes in our most mature and hypertrophic iMusc organoid model. We also chose 1 h to mimic endurance exercise and obtain a maximal response in signal over noise.^[^ ^21^, ^73^ ^]^

The mechanisms that underlie the rapid upregulation of mTOR signaling after exercise, and especially its concurrent upregulation of AMPK signaling, has been a long‐standing mystery.^[^ ^67^, ^74^ ^]^ While it is known how contraction‐induced calcium signaling and ATP depletion both can activate AMPK,^[^ ^75^, ^76^ ^]^ it is also well‐known that AMPK will inhibit mTOR signaling via the phosphorylation of TSC2 at Ser‐1387, which diminishes mTORC1 signaling.^[^ ^77^ ^]^ Moreover AMPK, activated by AICAR or phenformin, dephosphorylates Ser‐473 and Thr‐308 of AKT, thereby inhibiting its activity. This blockade of AKT by AMPK agonists consequently lowers insulin/IGF‐PI3K‐AKT‐mTORC1 signaling.^[^ ^78^ ^]^ On the other hand, it is also well‐known that exercise rapidly induces mTOR signaling without increasing insulin/IGF‐PI3K‐AKT signaling in the short‐term.^[^ ^79^, ^80^ ^]^ Thus, there must exist an alternative mechanism whereby exercise rapidly activates mTORC1, independently of the insulin/IGF‐PI3K‐AKT‐mTORC1 axis, but this long‐sought mechanism has hitherto remained a mystery. Previous studies showed that Lamtor1‐mTOR signaling promotes muscle strength.^[^ ^28^, ^70^ ^]^ Our results do suggest that exercise can rapidly induce protein expression of Lamtor1, thereby providing an alternative path via the amino acid‐sensitive Ragulator complex to activate mTORC1.^[^ ^81^, ^82^ ^]^ This provides a solution to the apparent paradox of concurrently activating AMPK and mTOR in a futile cycle through exercise, by suggesting that muscle Lamtor1‐AMPK is activated directly to quickly provide energy to the contracting muscle via catabolism,^[^ ^70^ ^]^ whereas Lamtor1‐mTORC1 is activated in the presence of amino acids to boost muscle growth and strength. Our findings thus position Lamtor1 as a central signaling hub with functions extending beyond exercise biology. As the core scaffold protein of the Ragulator complex, it indirectly governs cellular processes like autophagy and is emerging as a potential therapeutic target in other diseases such as cancer.^[^ ^83^, ^84^, ^85^ ^]^ This broader context underscores the fundamental importance of the mechanism we have identified. Coupled with our in vivo AAV9‐shRNA experiments and our analyses of aging human biopsy data (Figure 5I) and human exercise data (Figure 5J), our results demonstrate the physiological relevance of using 3D iMusc organoids to identify novel primary targets rapidly activated by physical exercise and muscle contractions. This is pertinent for the search of new muscle drugs for treating sarcopenic obesity and the metabolic syndrome, especially in an era of GLP‐1 analogs flooding the obesity drug market. There is a particular need for novel therapeutic strategies, such as AAV‐mediated gene therapy targeting regulators like Lamtor1, to counter the muscle wasting during such weight loss treatments.^[^ ^86^ ^]^ Despite these promising findings, we acknowledge the current model's lack of functional innervation and vascularization as core limitations and key areas for future improvement. Furthermore, direct histological and molecular benchmarking against human skeletal muscle tissue remains an important next step to further strengthen the model's validity, once matched clinical samples become accessible. Nevertheless, these limitations do not diminish the model's established value for studying intrinsic myocyte properties and rapid exercise‐like responses, as demonstrated in this study.

## 4 Experimental Section

### Myogenic Differentiation

The myogenic differentiation was based on Wnt pathway activation (CHIR99021) and BMP, TGF‐β pathway inhibition (LDN193189 & LY2157299), as published previously.^[^ ^87^, ^88^ ^]^ hESCs (H1 line) colonies were dissociated into single cells with accutase (Gibco) and seeded on Matrigel‐coated 12‐well plate at 30 000 cells well^−1^ in mTeSR1 medium (STEMCELL) supplemented with Y‐27632 (10 mm, Abcam). The somite differentiation was initiated with CHIR99021 (3uM, GSK3b inhibitor), LDN193189 (200 nm, BMP inhibitor), and LY2157299 (3uM, TGFβR inhibitor) for 4 days, followed by induction of dermomyotome formation for 2 days using a mixture of FGF2 (20 ng mL^−1^) and GSK3b inhibitors. All small‐molecule compounds were purchased from Selleck. On day 7, medium was switched to a myogenic culture medium supplemented with HGF (10 ng mL^−1^) and IGF‐1(20 ng mL^−1^) for 21 days, and the medium was changed every other day. On day 27–32, myoblasts derived from hESCs were expanded in the specialized growth medium supplemented with 20% FBS (BIOIND, Israel), FGF‐2 (20 ng mL^−1^), and 1% penicillin‐streptomycin (Gibco) at 37 °C and 5% CO_2_. Myoblasts (100% confluency) were induced to differentiate into myotube in a serum‐free medium (1% N2 supplement, Gibco) in DMEM (High glucose), 1% L‐glutamine (Gibco), and 1% penicillin‐streptomycin for 5–12 days to induce myotube formation.

### PDMS Molds and 3D Organoids Formation

6‐well plates were filled with 3 mL of liquid PDMS 184 Silicone Elastomer Kit (10:1, Dow Corning Corps., Michigan, USA). The PDMS was cured by incubating the plates at 37 °C for 8–12 h. Two 27G metal needles (serving as structural supports) were horizontally and symmetrically inserted into each PDMS‐cured well. One end of two 1 cm surgical silk threads (1‐0 diameter) was individually secured by two metal needles. Importantly, both silk threads (functioning as tendons) were aligned along the same horizontal direction. Each well was submerged in 70% ethanol within a sterile sealed container at room temperature for 0.5–3 h for sterilization. After triple rinsing with PBS containing 2% penicillin‐streptomycin, the PDMS plates underwent additional drying and sterilization via ultraviolet irradiation for 8–12 h.

For each 6‐well PDMS mold, the 5×10⁶ human myoblasts/gel mixture was prepared by pre‐mixing 300 µL of 1% N2 medium, 20 µL of thrombin (250 U mL^−1^, Sigma), 100 µL of Matrigel (Corning), and 6‐aminocaproic acid (6‐AA, 1 mg mL^−1^, Sigma) in the well. Subsequently, 200 µL of fibrinogen (20 µg mL^−1^, Sigma) was rapidly added to each well, with the entire procedure performed on ice. The cells/gel mixture was allowed to polymerize at room temperature for 15 min, followed by a 30‐min incubation at 37 °C with 5% CO_2_ to complete gelation. After 24 h of initial culture, the growth medium (supplemented with 1 mg mL^−1^ 6‐AA to maintain high‐density monolayers) was replaced with a differentiation medium containing 1% N2 Supplement. This differentiation medium was also supplemented with 6‐AA to inhibit fibrin degradation throughout the 10‐day differentiation period.

### Electrical Stimulation and Muscle Contractile Quantification

A programmable electrical stimulator (Model YC‐2, Chengdu Instrument Factory, China) was used to deliver biphasic stimulation to differentiated 3D iMusc organoids (days 10–14 post‐differentiation) in 6‐well plates. 3D iMusc organoids, either treated with drugs or used as untreated controls, were subjected to electrical pulses at 30 V, 10 ms pulse width, 1 Hz frequency (1000 ms interpulse interval) for 5 min (transcriptomic analysis) or 1 h (proteomic analysis). The contraction process of 3D iMusc organoids was recorded using a camera, and contractile displacement/length was calculated with ImageJ software and Microsoft Excel.

Isometric contractile quantification of 3D iMusc organoids was performed with modification as previously described.^[^ ^89^ ^]^ Briefly, samples were transferred into HBSS buffer supplemented with activating Ca^2+^solution (Beyotime, C0219) or Ringer's buffer (Solarbio, G0450) and incubated for 30 min at 37 °C. Each sample (0.8 cm in length) was immersed in a bath chamber maintained at 37 °C, secured to the hook of a motor arm (300C, Aurora Scientific, Canada) and a force transducer (Model Aurora Scientific 403A, Canada, force range: 5 mN; force resolution: 0.1 mN), then aligned with the force‐generating axis and stretched to ≈20% of muscle length. The Aurora Bi‐Phase stimulator was replaced by an external programmable stimulator (YC‐2, Chengdu Instrument Factory, China), the positive/negative platinum electrodes were placed in the bath chamber. For twitch contraction force measurement, the organoid was stimulated with electric pulses of 30 V, 10 ms width, 1 Hz frequency at 1000 ms intervals, constantly delivered for 20 s. Force signals from the transducers were recorded at 100 Hz with an A/D interface (604A, Aurora Scientific, Canada), and data were acquired using real‐time software (600A, Aurora Scientific). The contractile force of the first (1^st^) and the ninth (9^th^) Δpeak was further quantified with Microsoft Excel.

### Animals

The 4‐month‐old wild‐type C57BL/6J mice used in this study were purchased from SPF (BEIJING) BIOTECHNOLOGY CO, LTD. Mice were further randomly distributed into 2 groups to receive a single intramuscular injection of AAV9‐shCtrl or AAV9‐shLamtor1 (ViGene Biosciences, Jinan, China) at 1.65 × 10^11^v.g. particles at the volume of 30 µL in gastrocnemius muscles, as schematized in Figure 6A. Experimenters were blinded to group allocation during data collection. All animal procedures were approved by the Institute of Zoology and the Institute of Stem Cell and Regenerative Medicine, Chinese Academy of Sciences.

### Maximal Running Capacity Test

During the week prior to the maximal running capacity test, the mice were acclimated to the treadmill over five consecutive days by running for 1 minute at a speed of 8 m min^−1^, followed by 2 min at 9 m min^−1^, and finally for 7 min at 10 m min^−1^, all at a constant incline of 0°. The maximal running capacity test was then performed at a speed of 12 m min^−1^ until exhaustion, with a maximum running time set to 30 min.

### Maximal Hanging Time Test

Before conducting the hanging test, gently place the mice on the hanging apparatus to acclimate them to the experimental setup. The testing apparatus consists of a securely positioned horizontal grid located ≈30–50 cm above the ground. Once the mouse was placed on the apparatus, the timer was started. Record the time from when the mouse first grasps the grid until it either falls or releases its grip. To ensure the reliability of the results, each mouse undergoes three trials, with a recovery period of at least 1 h between trials.

### Grip Strength Test

A grip strength meter (Bioseb, Model: BIO‐GS3, France) was used to measure the four limbs grip strength of C57BL/6 mice. Pulled the mouse's tail backwards at a constant speed when it grasped the grid during a measurement. The peak force (g) was recorded after the mouse's paws detach from the grid. The strength meter was reset to 0 g after stabilization in each test. The order of mice tested every day was random, and all tests were operated by the same person to ensure the reliability of the data.

### Histology

Adipose tissues were fixed with 4% paraformaldehyde (PFA), dehydrated, embedded in paraffin, and cut into 7‐µm sections. The sections were stained with hematoxylin‐Eosin (HE) Stain Kit (G1120, Solarbio).

### Immunofluorescence Analysis

3D organoids were washed with PBS and fixed with pre‐cooled methanol at room temperature for 10 min. 2D myoblasts or myotubes were fixed with 4% PFA for 30 min at room temperature. Organoids were stained with the primary antibodies: MHC (AB2147781; DSHB, 2 mg mL^−1^), α‐actinin (A7811, Sigma, 1:500), Dystrophin (D8043, Sigma, 1:200), Ki67 (14‐5698‐82, Invitrogen, 1:200). 2D myoblasts or myotubes were stained with the primary antibodies: MyoD (sc‐377460; Santa Cruz, 1:200), MYOG (sc‐52903; Santa Cruz, 1:200), and PAX7(AB528428; DSHB, 5 ug mL^−1^), MHC (AB2147781; DSHB, 2 mg mL^−1^). The following secondary antibodies were conjugated with primary antibodies, Goat‐anti‐mouse Alexa Fluro 594 (A11005; Thermofisher; 1:500), Goat‐anti‐Rabbit Alexa Fluro 488 (A11008; Thermofisher; 1:500), Hoechst (62 249; Thermofisher; 1:1000) was used as a nuclear counter stain. Stained organoids were imaged with Andor Dragfly 200 Z‐axis phototangent imaging system.

### Muscle Fiber Typing and CSA

After skeletal muscles were harvested, they were fixed in isopentane pre‐cooled with liquid nitrogen for at least 30 min. Tissues were freeze‐embedded with OCT and sectioned with 10‐µm thickness. Before immunostaining, frozen slides were washed with PBS directly. Next, slides were treated with 0.1% Triton X‐100, 2%BSA/PBS at room temperature for 1 h. Slides were incubated with primary antibodies diluted in 0.1% Triton X‐100, 2%BSA, 5% goat serum/PBS at 4 °C overnight. The next morning, slides were washed with PBS and incubated with secondary antibodies diluted in 0.1% Triton X‐100, 2%BSA, 5% goat serum/PBS at room temperature and protected from light for 1 h. Slides were then washed with PBS and sealed with glycerin. For immunofluorescence assays, the following antibodies were used: anti‐myosin I (BA‐D5), anti‐myosin IIa (SC‐71), anti‐myosin IIb (BF‐F3) (all from DSHB, 1:50) and Laminin (L9393, Sigma, 1:500). Alexa Fluor secondary antibodies were used according to the manufacturer's instructions. Images were taken on a Nikon confocal microscope.

### Transmission Electron Microscopy

Samples were fixed with 2.5% (vol/vol) glutaraldehyde with Phosphate Buffer (PB) (0.1 m, pH 7.4), washed four times in PB. Then samples were postfixed with 1% (wt/vol) osmium tetraoxide in PB for 2 h at 4 °C, dehydrated through a graded ethanol series (30, 50, 70, 80, 90, 100%, 100%, 7 min each) into pure acetone (2×10 min). Samples were infiltrated in a graded mixtures (3:1, 1:1, and 1:3) of acetone and SPI‐PON812 resin (16.2 g SPI‐PON812, 10 g DDSA, and 8.9 g NMA), then changed pure resin. Finally, samples were embedded in pure resin with 1.5% BDMA and polymerized for 12 h at 45 °C, 48 h at 60 °C. The ultrathin sections (70 nm thick) were sectioned with microtome (Leica EM UC6), double‐stained by uranyl acetate and lead citrate, and examined by a transmission electron microscope (FEI Tecnai Spirit120kV).

### RNA‐Seq and GSEA Analysis

Samples (EPS‐ or EPS+) were disrupted and homogenized in TRIzol (Takara) using a rapid tissue crusher (CB‐prp‐01, Shanghai Cebo), total RNA was extracted for RNA‐seq. Sequencing libraries for RNA‐seq were provided by Novogene (Beijing).

GSEA was performed to define the significantly enriched pathways in EPS± organoids. The signaling pathway variation score and normalized enrichment scores (NES) were calculated by using the gene set variation analysis (GSVA) in R package and the GSEA tool (https://www.gsea‐msigdb.org/gsea/index.jsp), respectively. Gene sets with both p‐value and FDR q‐value <0.05 were considered as significantly enriched pathways.

### Label‐Free Quantitative Proteomics

Samples (EPS‐ or EPS+) were disrupted and homogenized in lysis buffer, and lysed on ice for 30 min. Protein solutions were isolated after centrifugation (13000 rpm, 20 min, and 4 °C), and the total protein concentration were quantified by BCA assays. Peptide extraction, trypsin digestion, TMT labeling, fractionation, LC‐MS/MS, and data acquisition, and data analysis, were as described in the previous publication.^[^ ^33^ ^]^

### Quantitative PCR

Quantitative real‐time PCR was performed using a LightCycler480 II sequence detection system (Roche Applied Science). RNA was extracted by TRIzol (Takara) and reverse transcribed with PrimeScript 286 RT (Takara), and Quantitative PCR was conducted by using KAPA SYBR FAST qPCR Kits (KAPA Biosystem) following the manufacturer's instructions. The primer sequences of human muscle‐specific markers were referred to in the previous article,^[^ ^87^ ^]^ respectively.

### Western Blotting

Samples were homogenized in RIPA Lysis buffer (Thermofisher, 89 901) with protease inhibitor cocktail (Thermofisher). All lysates were quantified by BCA protein assay (Thermofisher). 10 ug of protein from each sample were electrophoresed on SDS‐PAGE and transferred to nitrocellulose membranes (MECK Millipore). Blots were incubated with primary antibodies: MHC (AB2147781; DSHB), the other antibodies were purchased from Cell Signaling, LAMTOR (#8975T), pS6 (#5364S), p‐4EBP1 (#2855S), p‐AKT (#4060S), AKT (#2920S), p‐AMPK (#2535S), GAPDH (#2118), α‐tubulin (#3873), then probed with the secondary antibody anti‐mouse IgG HRP or anti‐rabbit IgG HRP (Thermofisher). HRP‐based detection was performed using an iBright FL1000 Imaging System (Invitrogen).

### Statistical Analysis

The experimental data were presented as Mean ± S.E.M. Statistical comparisons between two groups were performed using a two‐tailed unpaired Student's *t*‐test or Welch's *t*‐test (for unequal variances), with the two‐tailed Mann‐Whitney test applied for non‐parametric data. ANOVA with Tukey's post‐test was used for multiple group comparisons, and the Kruskal–Wallis test with Dunn's post‐test was used for non‐parametric data. Statistical significance was determined at the following p‐values: ^*^ *p *<0.05; **p <0.01; ***p <0.001. All experimental data analysis and statistical evaluation were performed using GraphPad Prism 9.0 and Microsoft Excel.

## Conflict of Interest

The authors declare no conflict of interest.

## Author Contributions

Z.Y., Z.J., X.L., and L.Z. contributed equally to this work. Z.Y. was primarily responsible for the experimental design, execution, and data analysis of genetic manipulation of organoids, animal studies, and electron microscopy. Z.J. was primarily responsible for the myogenic differentiation of hESCs, as well as the experimental design, execution, and data analysis for contractility assessment of organoids. L.Z., X.L., S.G., and P.W. helped with various experiments and mouse husbandry. Y.C. helped with quantitative RT‐PCR experiments. S.M. helped with Western blot experiments. L.L. helped with immunofluorescence experiments. Z.J. and Z.Y. wrote the manuscript. All authors discussed the data and approved the manuscript. N.S.‐C. supervised and coordinated the overall project.

## Supporting information



Supporting Information

Supplemental Video 1 Video S1: The 3D iMusc organoids contract synchronously in response to EPS (30 V,1000 ms, 1 HZ). Top: AdV‐Ctrl, bottom: AdV‐shLamtor1.

Supplemental Data

## Data Availability

The data that support the findings of this study are available in the supplementary material of this article.
