# Supplementary material for: Muscle Organoids Reveal Exercise‐Like Contractions Rapidly Promote Muscle Health Via Lamtor1's Signaling to Both AMPK and mTOR
Source: Adv Sci (Weinh). 2025 Oct 7;12(48):e05989. doi: 10.1002/advs.202505989 (PMC12752671; doi:10.1002/advs.202505989)

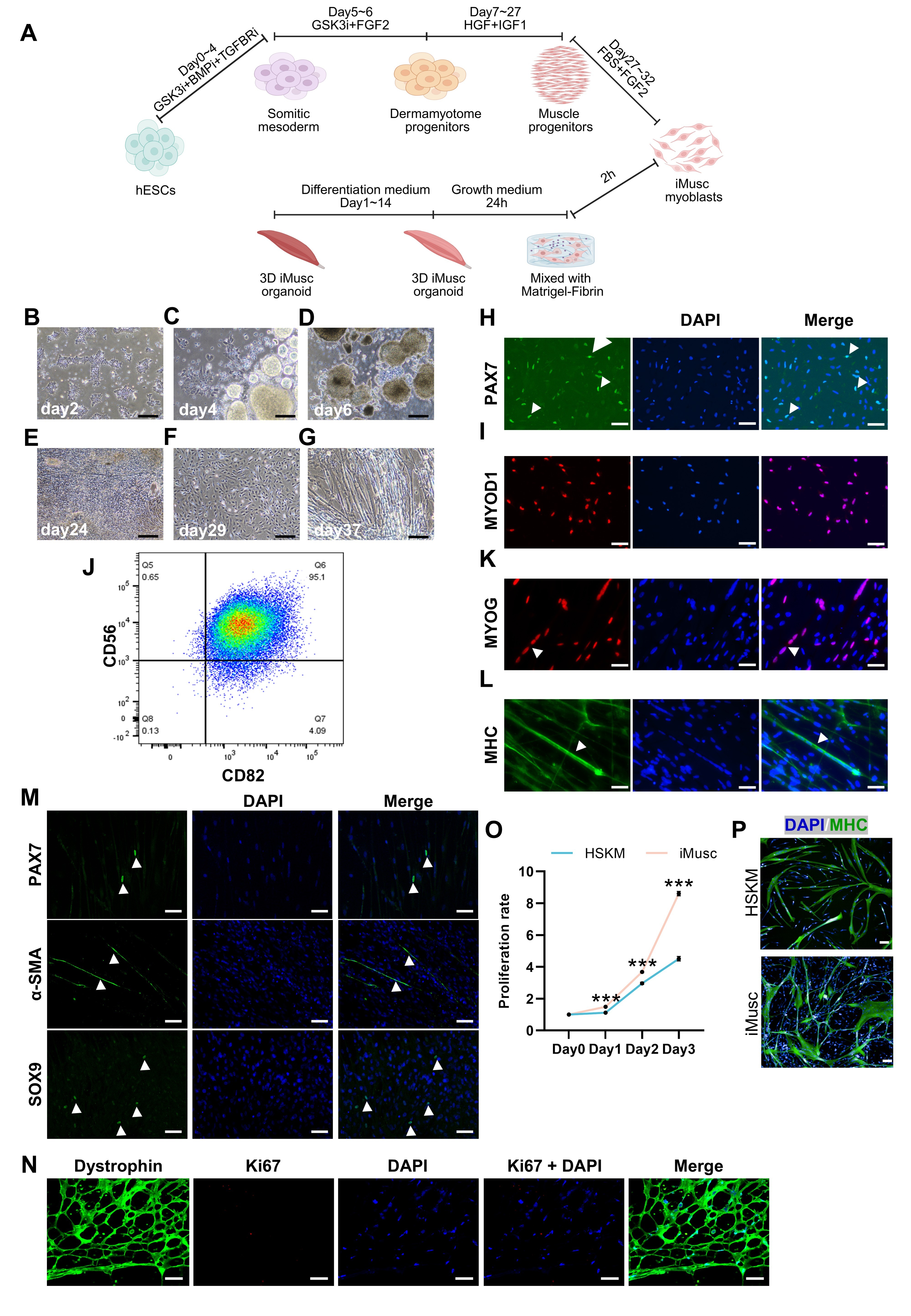


**Figure S1:** Characterization of induced skeletal muscle (iMusc) myoblasts and myotubes. A) Schematic diagram illustrating the cell source and construction of iMusc organoids. GSK3i: GSK3b inhibitor, CHIR99021; BMPi: BMP inhibitor, LDN193189; TGFβRi: TGFβR inhibitor, LY2157299; Growth medium: DMEM/F12 + 20%FBS + 1% Penicillin-Streptomycin (P/S); Differentiation medium: DMEM + 1%N2 supplement + 1%P/S. B - G) Representative phase contrast images of iMusc myoblasts and myotubes. Scale bars, 100 μm. H, I) Representative immunofluorescence (IF) staining of iMusc myoblasts for PAX7 (green) and MYOD1 (red). Scale bars, 50 μm. J) Flow cytometry analysis of iMusc myoblasts-specific surface markers. K, L) Representative IF staining of iMusc myotubes for MYOG (red) and MHC-fast (green). Scale bars, 20 μm. M) Representative IF staining of iMusc organoids for PAX7, α-SMA and SOX9. Scale bars, 75 μm. N) Representative IF staining of iMusc organoids for Ki67 and dystrophin. Scale bars, 50 μm. O) Quantitative comparison of proliferation rates between **human adult primary skeletal myoblasts (HSKM)** and **iMusc cells.** Data represent mean ± SEM from **4 independent biological replicates** (*n*=4). Statistical significance determined by two-tailed unpaired Student's t-test. P) Representative IF staining for MHC (green) in HSKM and iMusc myotubes. scale bars, 100 μm.


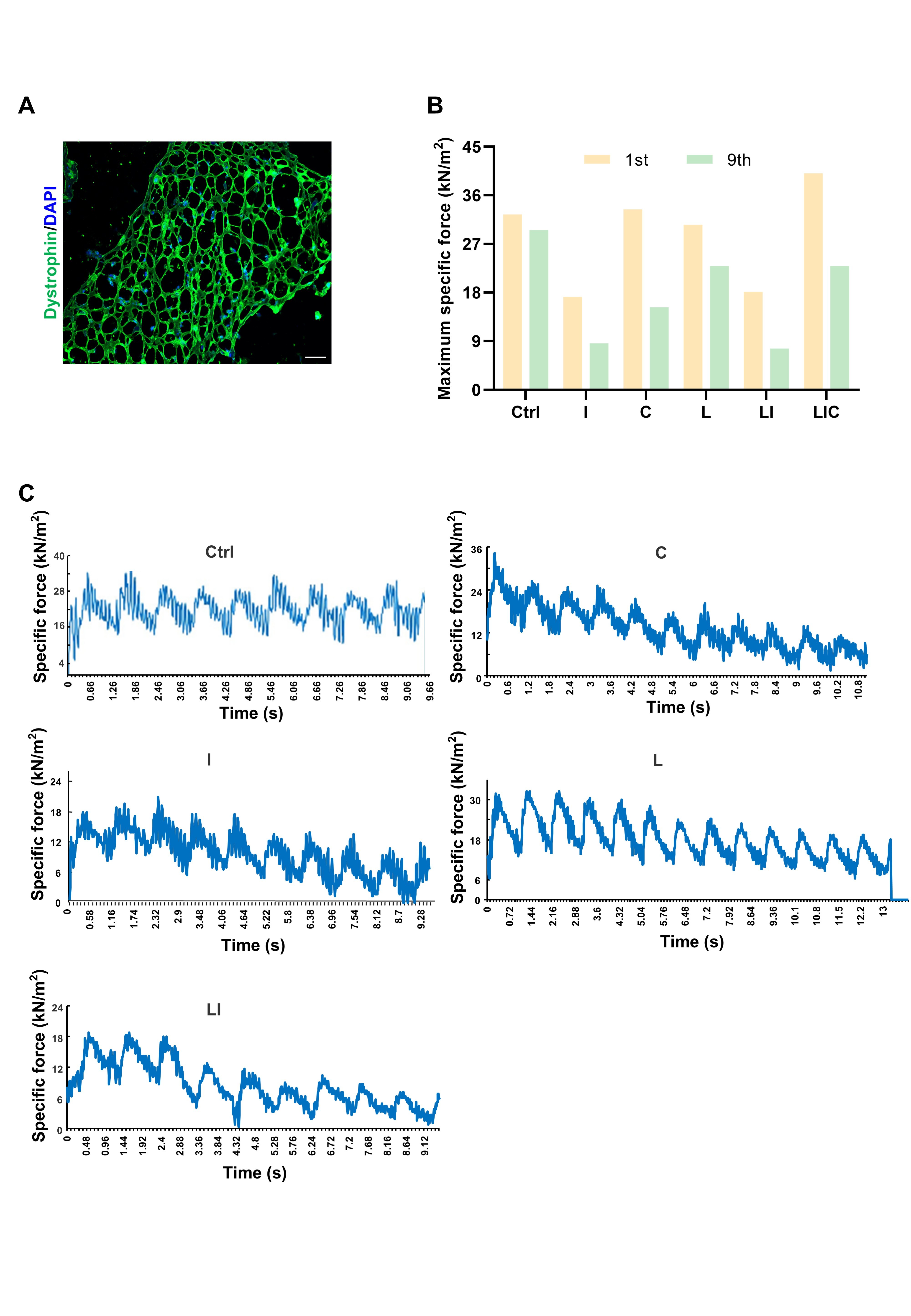


**Figure S2:** LIC enhances contraction activity in induced skeletal muscle (iMusc) organoids. A) Representative immunofluorescence (IF) staining image of dystrophin (green) in 3D iMusc organoids for CSA analysis. Scale bars, 50 μm. B) Quantification of maximum specific force (kN/m^2^) at the first and the ninth contraction peaks in Ctrl and drug-treated 3D iMusc organoids. C) Representative specific twitch force (kN/m^2^) traces of drug-treated 3D iMusc organoids. EPS: 1 Hz, 30 V, 10 ms pulse width, 1000 ms inter-pulse interval.


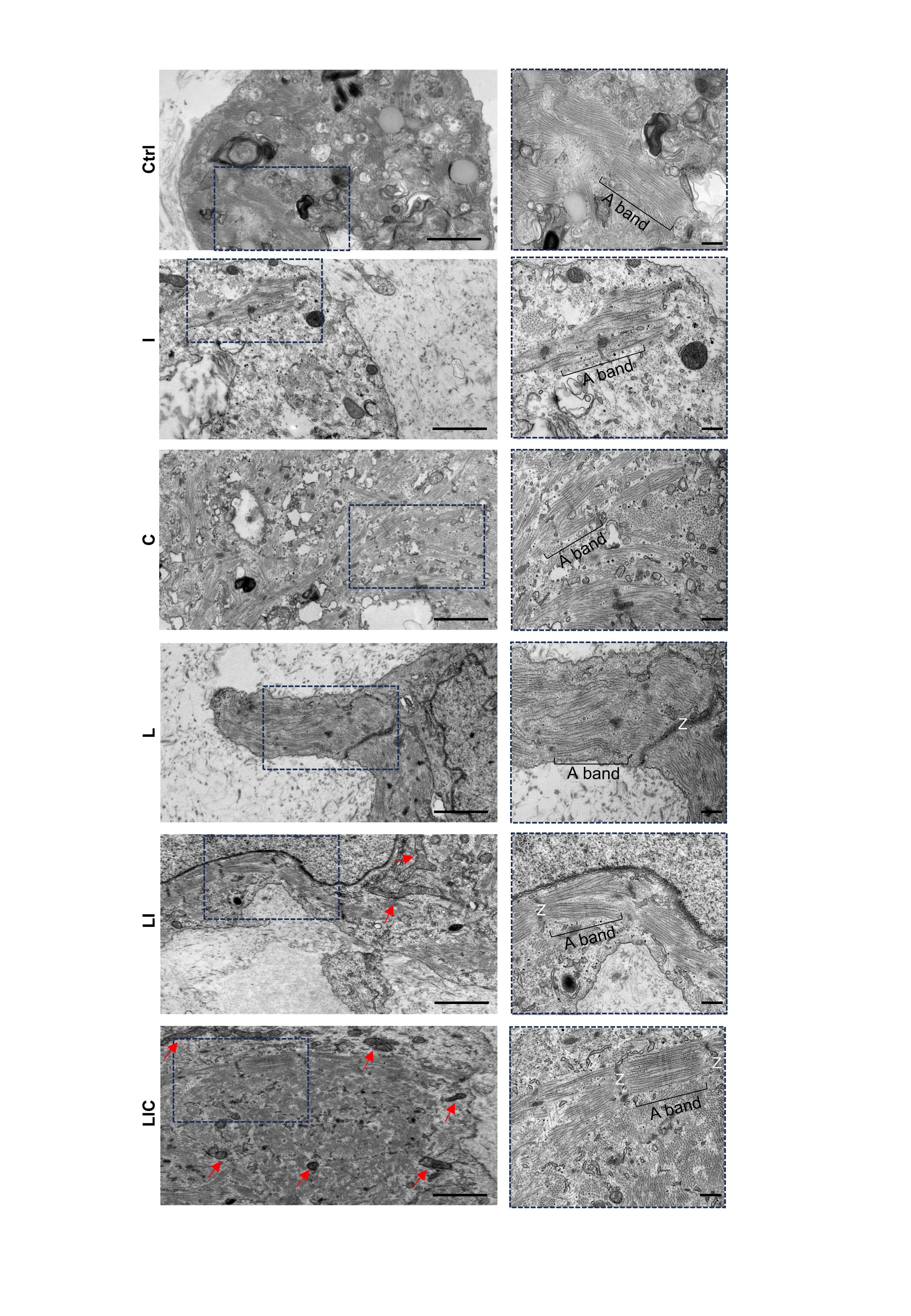


**Figure S3:** LIC promotes maturation and hypertrophy in induced skeletal muscle (iMusc) organoids.

Representative transmission electron microscopy images of sarcomeres and mitochondria in 3D iMusc organoids treated with Ctrl (control), C (calcitriol), I (IGF-1), L (LY2157299), LI (LY2157299 + IGF-1), LIC (LY2157299 + IGF-1 + calcitriol). The red arrows point to mitochondria. Scale bars: 2 μm; enlarged view scale bars, 500 nm.


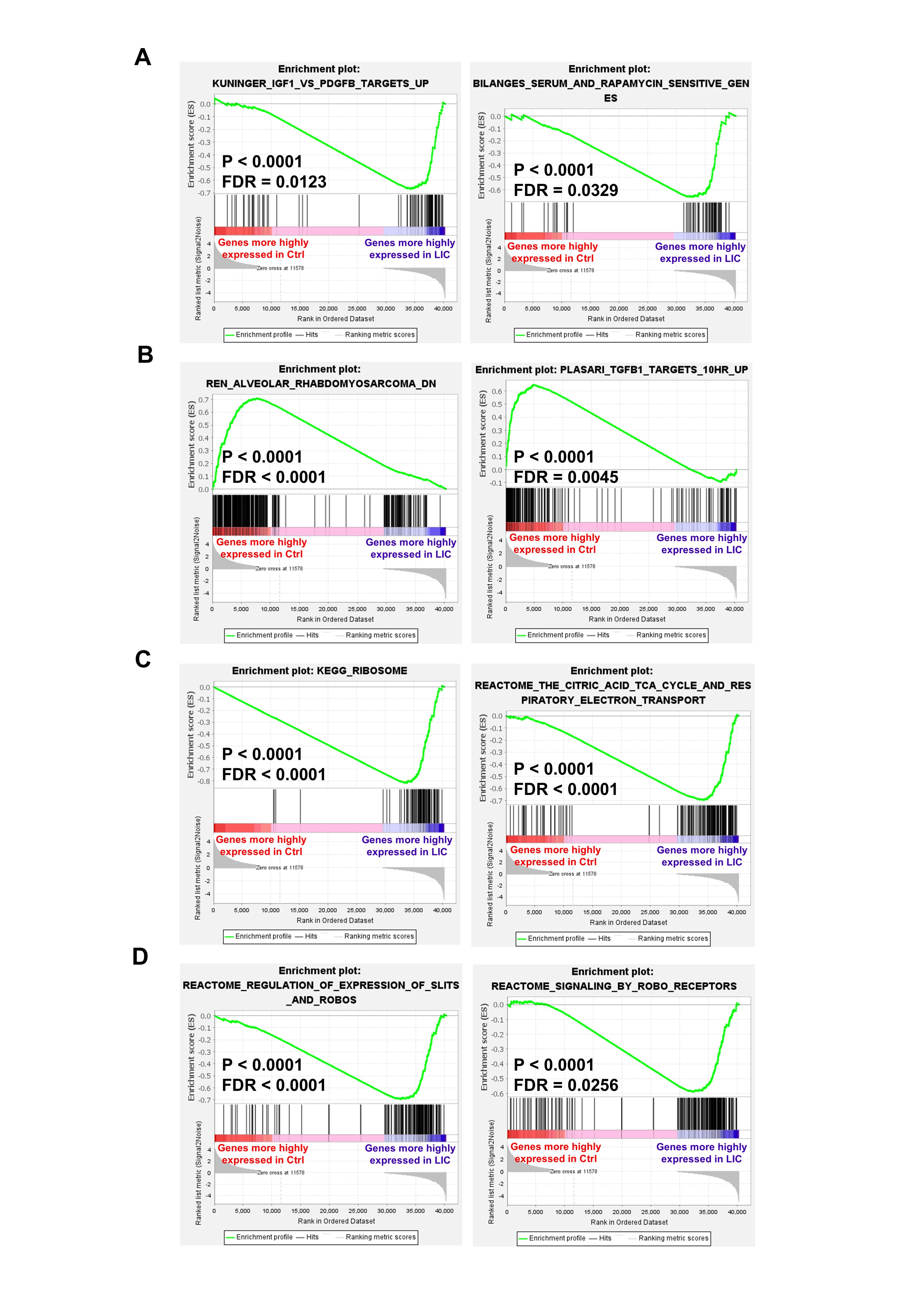


**Figure S4:** LIC activates IGF signaling and suppresses TGFβ signaling in induced skeletal muscle (iMusc) organoids. A - D) GSEA plots from RNA-seq data showing upregulated signatures (IGF signal, ribosomes, mitochondrial oxidative phosphorylation, and Robo-Slit signaling) and downregulated signatures (TGFβsignaling) in LIC-treated 3D iMusc organoids.


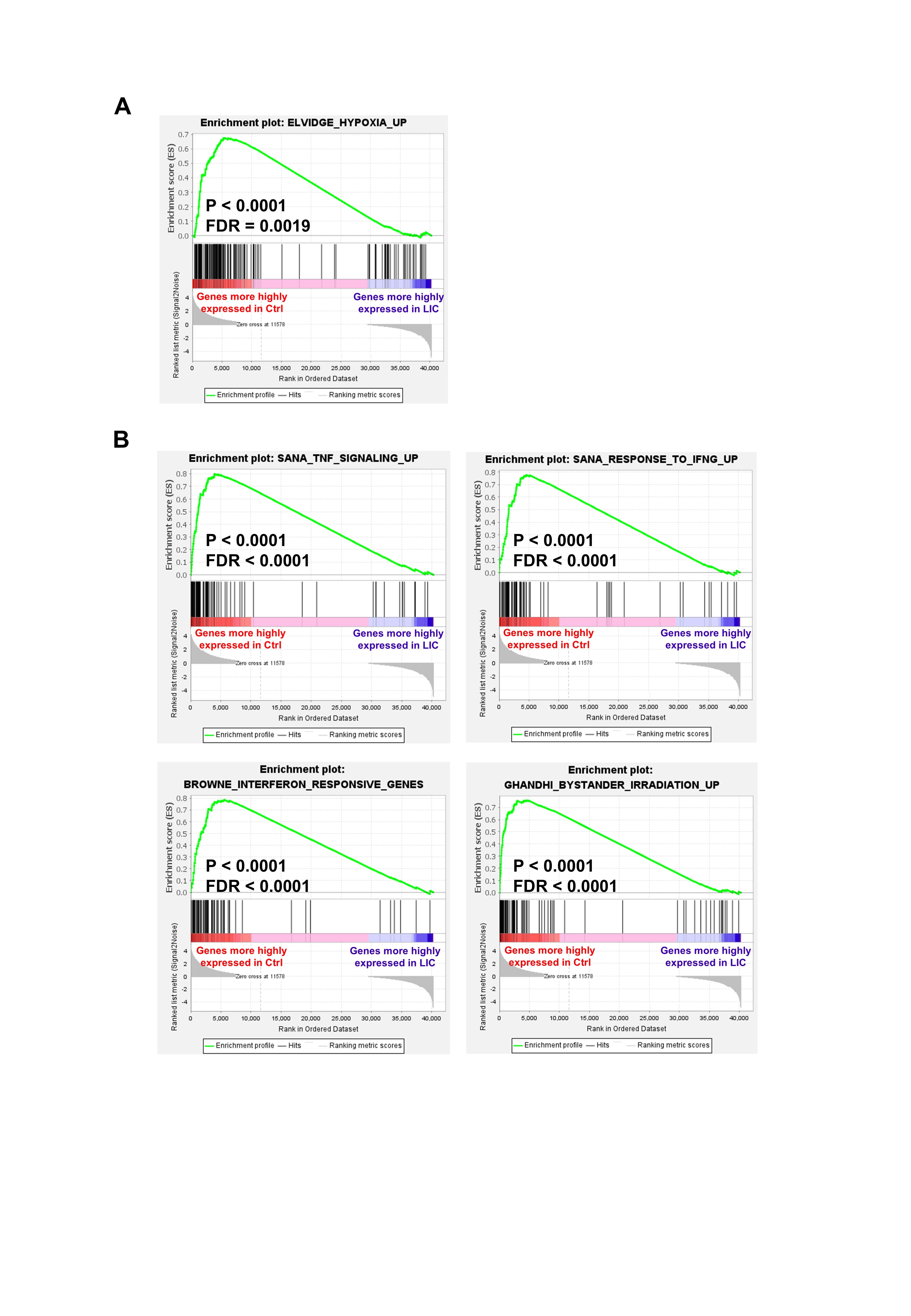


**Figure S5:** LIC reduces hypoxia and inflammation stress signatures in induced skeletal muscle (iMusc) organoids.

(A, B) GSEA plots from RNA-seq data showing downregulated signatures (hypoxia and inflammation stress) in LIC-treated 3D iMusc organoids.


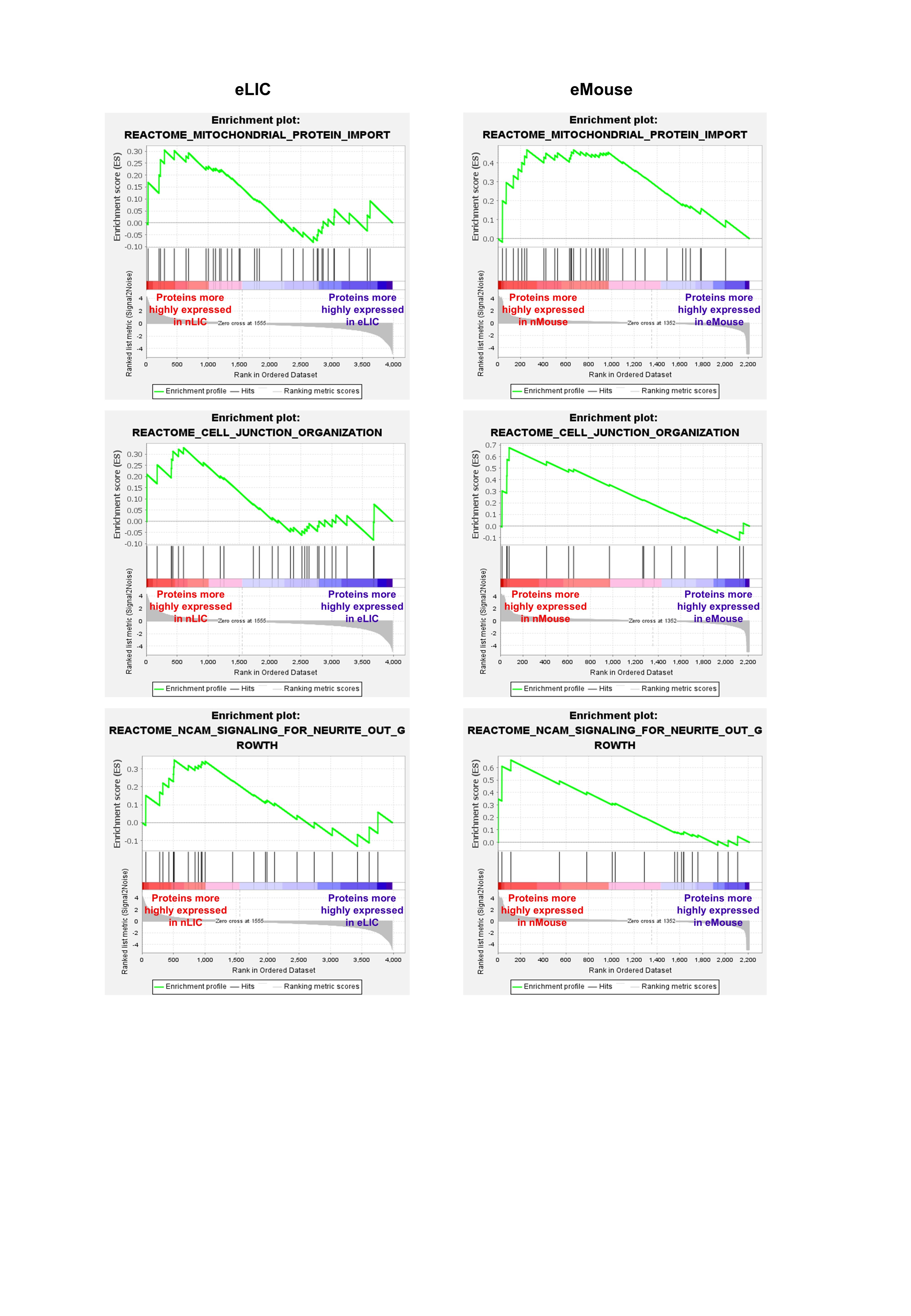


**Figure S6:** Electrical stimulation induces conserved transcriptomic changes in LIC-treated induced skeletal muscle (iMusc) organoids and mouse muscles.

PSEA plots showing overlapped signatures in eLIC-treated 3D iMusc organoids (left) and eMouse muscles (right).


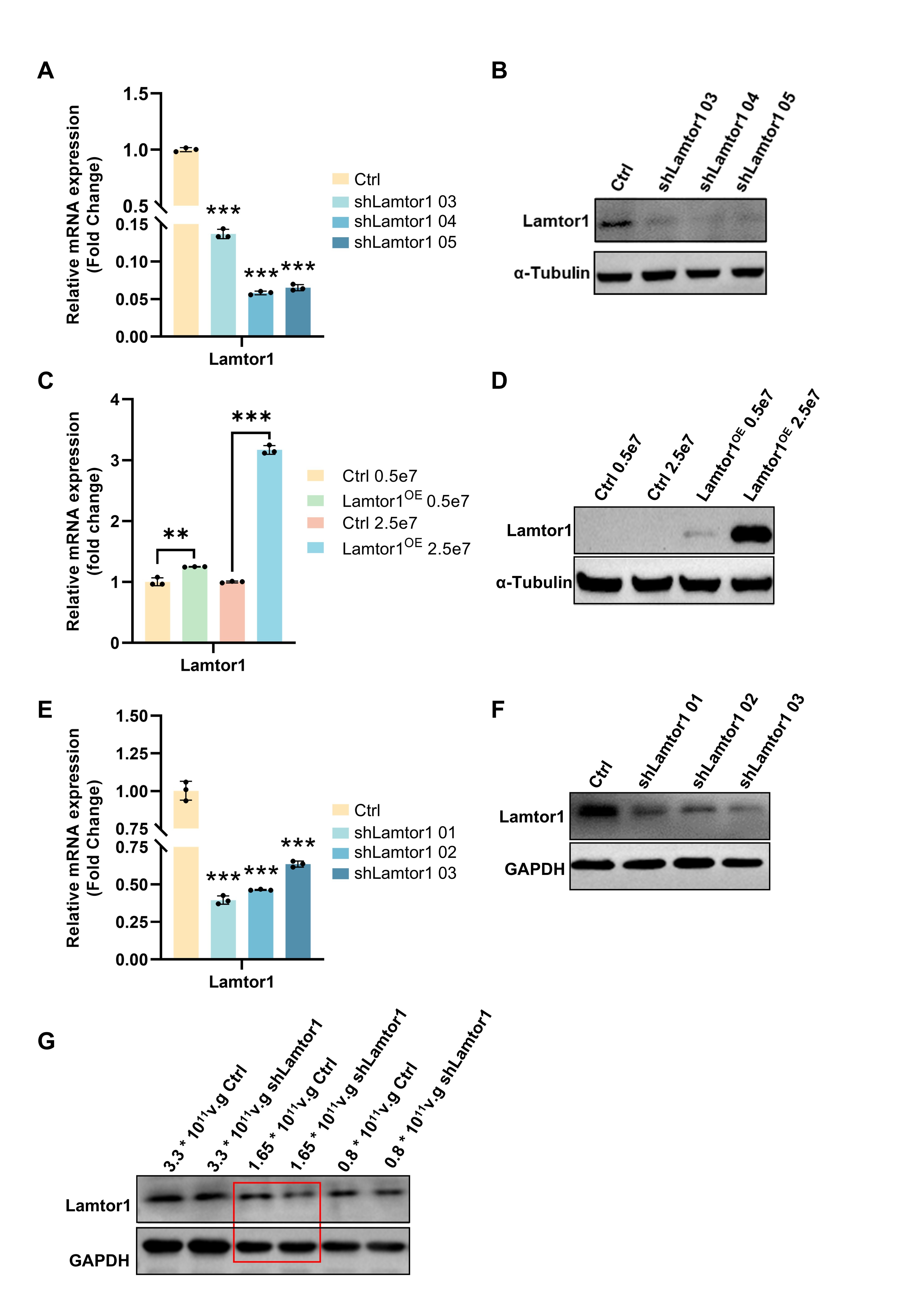


**Figure S7:** Validation of viral vector-mediated Lamtor1 knockdown and overexpression efficiency *in vitro* and *in vivo*. A, B) The AdV-shRNA sequence selected for its highest knockdown efficiency. A) Quantitative RT-PCR analysis. Data are shown as Means ± SEM (*n*=3 independent experiments). Statistical significance determined by one-way ANOVA with post-hoc Tukey's test; **P*<0.05, ***P*<0.01, ****P*<0.001. B) Representative western blots. α-tubulin served as the loading control. C, D) The AdV-OE-Lamtor1 titer screening for optimal overexpression efficiency. C) Quantitative RT-PCR analysis. Data are shown as Means ± SEM (*n*=3 independent experiments). Statistical significance determined by two-tailed unpaired Student's t-test.; **P*<0.05, ***P*<0.01, ****P*<0.001. D) Representative western blots. α-tubulin served as the loading control. E, F) The AAV9 shRNA sequence selected for its highest knockdown efficiency. E) Quantitative RT-PCR analysis. Data are shown as Means ± SEM (*n*=3 independent experiments). Statistical significance determined by one-way ANOVA with post-hoc Tukey's test; **P*<0.05, ***P*<0.01, ****P*<0.001. F) Representative western blots. α-tubulin served as the loading control. G) Representative western blots of AAV9-shRNA dosage in gastrocnemius of C57BL/6 mice. GAPDH served as the loading control.


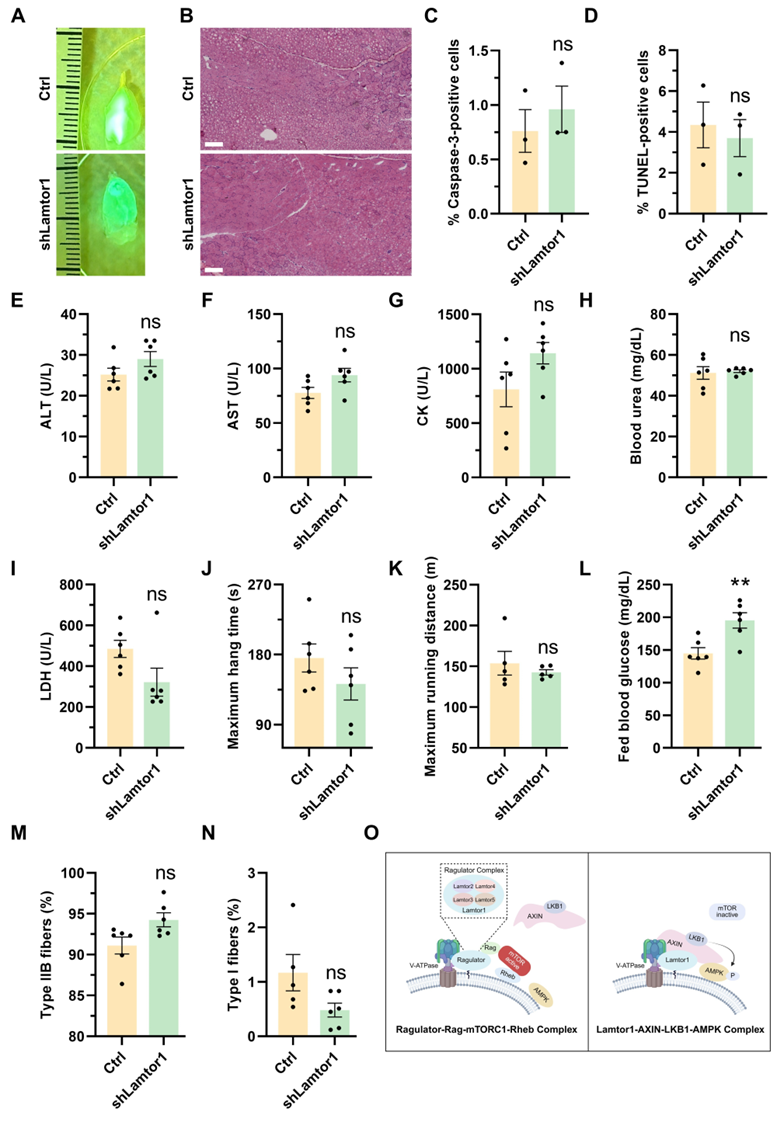


**Figure S8:** shLamtor1 mice exhibit elevated fed blood glucose levels and impaired exercise capacity. A) Representative images of isolated gastrocnemius muscles from control (Ctrl) and shLamtor1 mice. EGFP fluorescence confirms AAV9-mediated transduction. B) H&E staining of gastrocnemius sections. Scale bars, 200 µm. C) Quantification of Caspase-3-positive cells. Data are shown as Means ± SEM (*n*=3). Statistical significance determined by two-tailed unpaired Student's t-test; ns: not significant, **P*<0.05, ***P*<0.01, ****P*<0.001. D) Percentage of TUNEL-positive cells. Data are shown as Means ± SEM (*n*=3). Statistical significance determined by two-tailed unpaired Student's t-test; ns: not significant, **P*<0.05, ***P*<0.01, ****P*<0.001. E – I) Serum markers of tissue damage: (E) ALT (alanine aminotransferase), (F) AST (aspartate aminotransferase), and (G) CK (creatine kinase), (H) Urea, and (I) LDH (lactate dehydrogenase). Data are shown as Means ± SEM (*n*=6). Statistical significance determined by two-tailed unpaired Student's t-test; ns: not significant, **P*<0.05, ***P*<0.01, ****P*<0.001. J) Maximum hang time in Ctrl and shLamtor1 mice. Data are shown as Means ± SEM (*n*=6). Statistical significance determined by two-tailed unpaired Student's t-test; ns: not significant, **P*<0.05, ***P*<0.01, ****P*<0.001. K) Maximum running distance in Ctrl and shLamtor1 mice. Data are shown as Means ± SEM (*n*=5). Statistical significance determined by two-tailed unpaired Student's t-test; ns: not significant, **P*<0.05, ***P*<0.01, ****P*<0.001. L) Fed blood glucose levels in Ctrl and shLamtor1 mice. Data are shown as Means ± SEM (*n*=6). Statistical significance determined by two-tailed unpaired Student's t-test; ns: not significant, **P*<0.05, ***P*<0.01, ****P*<0.001. M) Quantification of type IIB myofiber area percentage in gastrocnemius muscle. Data are shown as Means ± SEM (*n*=6). Statistical significance determined by two-tailed Mann-Whitney test; ns: not significant, **P*<0.05, ***P*<0.01, ****P*<0.001. N) Quantification of type I myofiber area percentage in gastrocnemius muscle. Data are shown as Means ± SEM (Ctrl, *n*=5; shLamtor1, *n*=6). Statistical significance determined by two-tailed unpaired Student's t-test; ns: not significant, **P*<0.05, ***P*<0.01, ****P*<0.001. O) The dual role of Lamtor1 in coordinating Ragulator-mTORC1-mediated anabolic and Lamtor1-AMPK-mediated catabolic signaling pathways.

This study develops a novel 3D human skeletal muscle organoid platform to study the immediate molecular effects of contraction. The model uncovers rapid genomic and proteomic responses, resolving a fundamental paradox by demonstrating how Lamtor1 coordinately activates both AMPK and mTORC1. This mechanism presents Lamtor1 as a compelling target for therapeutic exercise mimicry.


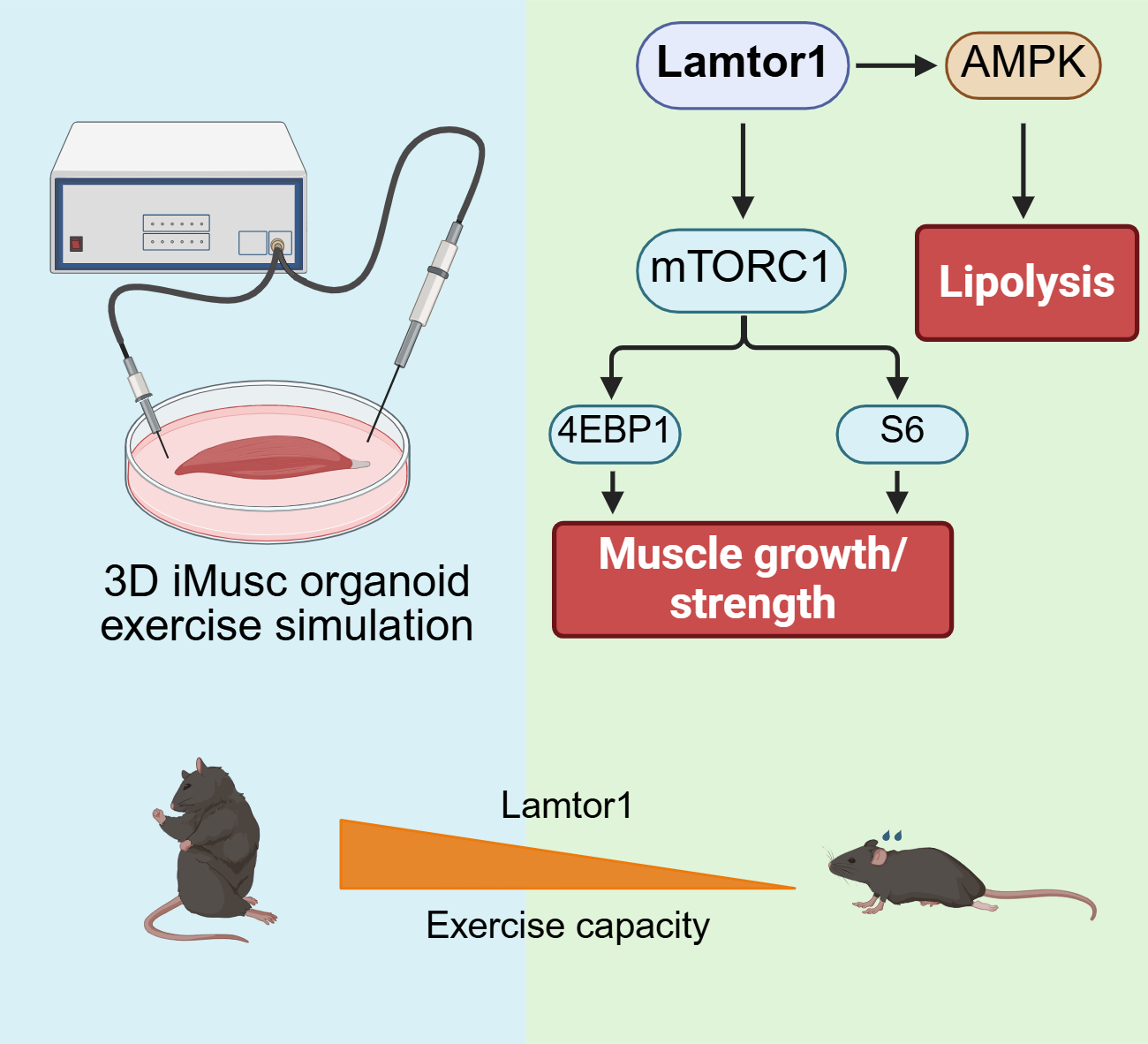

Supplement: Supplementary file 1 — Supporting Information [file ADVS-12-e05989-s001.docx]
